# Interleukin-1 promotes autoimmune neuroinflammation by suppressing endothelial heme oxygenase-1 at the blood–brain barrier

**DOI:** 10.1007/s00401-020-02187-x

**Published:** 2020-07-11

**Authors:** Judith Hauptmann, Lisa Johann, Federico Marini, Maja Kitic, Elisa Colombo, Ilgiz A. Mufazalov, Martin Krueger, Khalad Karram, Sonja Moos, Florian Wanke, Florian C. Kurschus, Matthias Klein, Silvia Cardoso, Judith Strauß, Subhashini Bolisetty, Fred Lühder, Markus Schwaninger, Harald Binder, Ingo Bechman, Tobias Bopp, Anupam Agarwal, Miguel P. Soares, Tommy Regen, Ari Waisman

**Affiliations:** 1 Institute for Molecular Medicine, University Medical Center of the Johannes Gutenberg-University Mainz, Mainz, Germany; 2 Center of Thrombosis and Hemostasis Mainz (CTH), University Medical Center of the Johannes Gutenberg-University Mainz, Mainz, Germany; 3 Institute for Medical Biostatistics, Epidemiology and Informatics (IMBEI), University Medical Center of the Johannes Gutenberg-University Mainz, Mainz, Germany; 4 Anatomical Institute, University of Leipzig, Leipzig, Germany; 5 Institute for Immunology, University Medical Center of the Johannes Gutenberg-University Mainz, Mainz, Germany; 6 Research Center for Immunotherapy (FZI), University Medical Center of the Johannes Gutenberg University Mainz, Mainz, Germany; 7 Instituto Gulbenkian de Ciência, Oeiras, Portugal; 8 Institute for Neuroimmunology and Multiple Sclerosis Research, University Medical Center Göttingen, Göttingen, Germany; 9 Institute for Experimental and Clinical Pharmacology and Toxicology, University of Lübeck, Lübeck, Germany; 10 Institute of Medical Biometry and Statistics, Faculty of Medicine and Medical Center, University of Freiburg, Freiburg, Germany; 11 Nephrology Research and Training Center, School of Medicine, University of Alabama at Birmingham, Birmingham, AL USA; 12 Present Address: Immunology, Infectious Diseases and Ophthalmology (I2O) Discovery and Translational Area Roche Innovation Center, Basel, Switzerland; 13 Present Address: Department of Dermatology, Heidelberg University Hospital, 69120 Heidelberg, Germany

**Keywords:** Blood–brain barrier, Interleukin-1, Autoimmunity, Experimental autoimmune encephalomyelitis (EAE), Heme oxygenase-1 (HO-1)

## Abstract

The proinflammatory cytokine interleukin 1 (IL-1) is crucially involved in the pathogenesis of multiple sclerosis (MS) and its animal model experimental autoimmune encephalomyelitis (EAE). Herein, we studied the role of IL-1 signaling in blood–brain barrier (BBB) endothelial cells (ECs), astrocytes and microglia for EAE development, using mice with the conditional deletion of its signaling receptor IL-1R1. We found that IL-1 signaling in microglia and astrocytes is redundant for the development of EAE, whereas the IL-1R1 deletion in BBB-ECs markedly ameliorated disease severity. IL-1 signaling in BBB-ECs upregulated the expression of the adhesion molecules Vcam-1, Icam-1 and the chemokine receptor Darc, all of which have been previously shown to promote CNS-specific inflammation. In contrast, IL-1R1 signaling suppressed the expression of the stress-responsive heme catabolizing enzyme heme oxygenase-1 (HO-1) in BBB-ECs, promoting disease progression via a mechanism associated with deregulated expression of the IL-1-responsive genes *Vcam1*, *Icam1* and *Ackr1* (Darc). Mechanistically, our data emphasize a functional crosstalk of BBB-EC IL-1 signaling and HO-1, controlling the transcription of downstream proinflammatory genes promoting the pathogenesis of autoimmune neuroinflammation.

**Electronic supplementary material:**

The online version of this article (10.1007/s00401-020-02187-x) contains supplementary material, which is available to authorized users.

## Introduction

The inflammatory disease multiple sclerosis (MS) and its animal model experimental autoimmune encephalomyelitis (EAE) are characterized by peripheral immune cell infiltration into the central nervous system (CNS), which ultimately leads to demyelination and axonal damage in the brain and spinal cord [38]. Immune cell infiltration in MS/EAE is a consequence of focal blood–brain barrier (BBB) breakdown, which enables the uncontrolled influx of peripheral cells and molecules. The BBB is a tightly regulated vascular barrier, composed of specialized endothelial cells (ECs), connected through intercellular tight junctions and adherens junctions, thereby controlling traffic of cells and molecules across the BBB [79]. Disorganization of these junctional proteins together with upregulation of cell adhesion molecules, such as intercellular adhesion molecule-1 (Icam-1) and vascular adhesion molecule-1 (Vcam-1), is associated with peripheral cell adhesion and migration into the CNS in MS and EAE [7].

Interleukin (IL)-1 is an important inflammatory cytokine, strongly implicated as an effector molecule in MS and EAE [23]. The IL-1α and IL-1β isoforms signal through a common receptor complex, composed of the IL-1 receptor type 1 (IL-1R1) and its accessory protein (IL-1RAcP). The downstream signaling cascade induces activation of the mitogen-activated protein kinases (MAPK) and mobilization of the nuclear factor kappa-light-chain-enhancer of activated B cells (NF-κB) family of transcription factors, which trigger the transcription of proinflammatory genes [74]. Macrophages, dendritic cells and neutrophils are the major sources of IL-1β production in EAE [22, 37, 42, 80]. Levesque and colleagues identified neutrophils and monocyte-derived macrophages as the primary cell types expressing IL-1β in EAE upon their transmigration into the spinal cord parenchyma [39]. In contrast to IL-1α, mice deficient for *Il1b* are resistant to EAE, identifying IL-1β as a critical mediator of EAE development [39, 61]. Likewise, *Il1r1*-deficient mice are also resistant to EAE, which correlates with the failure to induce autoreactive T helper (T_H_) 17 cells [19, 54, 61, 77]. Importantly, mice with a global *Il1r1* deficiency are less susceptible to EAE, as compared to mice with a T cell-specific *Il1r1* deletion, suggesting that other cell types may play a pathogenic role in response to IL-1 in EAE [61]. Furthermore, using bone marrow chimeric mice, it has been shown that IL-1R1 signaling on radiation-resistant cells promotes EAE development [39, 55]. Together, these results identify a pathogenic role for IL-1β signaling in EAE that goes beyond the induction of autoreactive CD4 T cells.

Cellular targets of IL-1 within the CNS have been controversially debated. On one hand, BBB endothelial cells (BBB-ECs) have been reported to be the major cell type expressing IL-1R1 [35, 39, 45], thereby driving leukocyte recruitment and compromising BBB integrity [16, 63]. Astrocytes and microglia have also been found to respond to IL-1β and were, therefore, suggested to be key players during neuroinflammation [11, 15, 57, 64]. IL-1β induces a rapid proinflammatory response in astrocyte cultures, leading to the expression of cytokines, chemokines, adhesion molecules, and matrix metalloproteinases [6, 36, 78, 81]. Furthermore, IL-1β induces astrocytic expression of angiogenic factors, including vascular endothelial growth factor A (VEGF-A), which is involved in vessel plasticity and angiogenesis, thereby representing an important driver of BBB disruption in EAE [9, 75].

A comprehensive understanding in mechanisms of IL-1-mediated signaling in CNS-resident cells, including BBB-ECs, astrocytes and microglia, during the course of EAE is far beyond completed. To study the cell type-specific role of IL-1 signaling, we made use of conditional gene targeting to obtain novel mouse strains, where the IL-1R1 is specifically deleted in different CNS-resident cell populations. In this study, we show that the deletion of IL-1R1 in BBB-ECs reduced EAE severity, whereas IL-1 signaling in astrocytes or microglia was redundant for EAE development. Differential gene expression analysis of BBB-ECs at the preclinical stage of EAE identified that IL-1 signaling suppressed the expression of heme oxygenase-1 (HO-1), a core effector molecule that prevents tissue damage under stress conditions [53]. We found that the deletion of HO-1 specifically in BBB-ECs resulted in an enhanced EAE disease. Targeting the expression of HO-1 exclusively to BBB-ECs in vivo revealed its protective function specifically on BBB-ECs. Moreover, our findings suggest a functional crosstalk between IL-1 signaling and HO-1, counter-regulating the expression of adhesion molecules that promote the pathogenesis of EAE. Our results, therefore, identify BBB-EC HO-1 as a potential target for therapeutic interventions in the course of neuroinflammatory processes associated with BBB breakdown.

## Materials and methods

### Mice

*Il1r1*^*fl/fl*^ mice [2] were crossed to *Gfap-Cre* [10], *Cx3cr1-Cre*^*ERT2*^ [85], *Slco1c1-Cre*^*ERT2*^ mice [65], to obtain IL-1R1^GFAP^, IL-1R1^CX3CR1^, IL-1R1^SLC^. Cre-negative *Il1r1*^*fl/fl*^ (IL-1R1^WT^) littermates and complete *Il1r1* deficient mice (IL-1R1^−/−)^ [60, 61] were used as control animals.

RiboTag (*RPL22*^*HA*^) mice [66] were crossed to the IL-1R1^SLC^ mouse strain to obtain IL-1R1/HA^SLC^ mice, or crossed to the *Slco1c1-Cre*^*ERT2*^ mice (HA^SLC^ mice), which were used as controls. Conditional *ROSA-flox-human-HMOX-1* overexpression mice (*hHO-1oe*^*fl/fl*^) [13], were bred to *Slco1c1-Cre*^*ERT2*^ mice to obtain hHO-1oe^SLC^ mice. Cre-negative *hHO-1oe*^*fl/fl*^ (hHO-1oe^WT^*)* were used as control mice. *HO-1*^*fl/fl*^ (B6J.129P2-Hmox1 < tm1Mym > , purchased from RIKEN) were crossed to *Slco1c1-Cre*^*ERT2*^ mice to obtain HO-1^SLC^ mice, whereas Cre-negative *HO-1*^*fl/fl*^ (HO-1^WT^) mice were used as controls. Mice were housed and bred under specific pathogen-free (SPF) conditions, with a 12 h light/dark cycle and unlimited access to food and water at the University Medical Center Mainz. For all experiments, 10–14-week-old age- and sex-matched littermate animals were used, in accordance with the guidelines of the central animal facility institution (Translational Animal Research Center, University of Mainz).

### Tamoxifen treatment

To induce Cre expression in BBB-ECs, 5–7-week-old *Slco1c1-Cre*^*ERT2*^-carrying mice and their Cre-negative littermate controls were intraperitoneally injected with tamoxifen (2 mg in olive oil containing 5% ethanol) on 5 consecutive days. *Cx3cr1-Cre*^*ERT2*^ mice and their Cre-negative littermates received two subcutaneous tamoxifen injections (2 mg in olive oil containing 5% ethanol) on postnatal days 12 and 14.

### EAE induction and scoring

Mice were immunized subcutaneously at the tail base with 50 μg MOG_35-55_ peptide (GenScript) emulsified in complete Freund’s adjuvant (CFA, BD Biosciences) supplemented with 10 mg/ml *Mycobacterium tuberculosis* H37RA (BD Biosciences). In addition, mice were intraperitoneally injected with 100 ng of pertussis toxin (PTx) (List Biological Labs) on day 0 and day 2 post-immunization. Mice were observed daily, to monitor body weight and EAE clinical symptoms on a scale from 0 to 4 as follows: 0, no disease; 0.5: limb tail; 1: paralyzed tail; 1.5: weakened righting reflex; 2: no righting reflex; 3: partial paralysis of hind legs; 3.5: paralysis of one hind leg; 4: paralysis of both hind legs.

### Lymphocyte cell isolation from CNS and lymphoid tissue

Mice were sacrificed with CO_2_ prior to dissection of CNS and lymphoid organs. For CNS lymphocyte isolation, CNS tissue was dissected from mice transcardially perfused with 0.9% NaCl solution (Sigma-Aldrich) and digested with 2 mg/ml collagenase II (Gibco) and 2 μg/ml DNase I (Roche) for 20 min at 37 °C and subsequently homogenized with a 18-G needle. Cells were then separated using a 70–37–30% Percoll (Sigma-Aldrich) gradient centrifugation for 40 min, 500×*g* at 10 °C. Cells at the 70/37% interphase were carefully collected and washed in PBS containing 2% FCS (Thermo Scientific) (PBS/FCS) prior to 10 min centrifugation at 500×*g*. To specifically characterize MOG-specific T cells, cells were plated in 96-well U-bottom plates and re-activated with 20 μg/ml MOG_35-55_ peptide and brefeldin A (Sigma-Aldrich) for 6 h at 37 °C. Afterwards, cells were harvested and stained for flow cytometry analysis. T cell expression of CD154 (CD40L) was assessed as indication of recent activation, serving as a surrogate marker for MOG antigen specificity. CD40L^+^ cells were further analyzed for their cytokine expression. For peripheral lymphoid cell isolation, lymph nodes and spleen were mechanically dissociated in PBS/FCS and filtered through a 40 μm cell strainer. Erythrocytes were removed by Ammonium-chloride-potassium lysis (1 M Tris, 1 M MOPS, 20 mM EDTA, 2% SDS, pH 7.7) and lymphoid cells afterwards washed with PBS/FCS. Cells were counted and equal amounts were re-activated in vitro in the presence of MOG_35-55_ peptide to assess the proportion of MOG-specific T cells as indicated above. For IL-1β detection, cells were incubated for 4 h in the presence of 2 μM monensin (BioLegend) and 500 ng/ml LPS (Sigma),

### Endothelial cell isolation for flow cytometry

For CNS EC-isolation, mice were sacrificed and transcardially perfused as described above. The dissected CNS tissue was digested with 2 mg/ml papain (Sigma-Aldrich) solution containing 40 μg/ml DNase I for 30 min at 37 °C. During incubation, tissue was mechanically homogenized using the gentleMACS™ Dissociator (Miltenyi). The resulting cell suspension was filtered through a 70 μm cell strainer and centrifuged with a 20% Percoll gradient for 30 min, 300×*g* at 15 °C. The pellet, containing a mix of CNS cells, was used for flow cytometry staining.

### Flow cytometry analyses

Before antibody staining, Fc receptors were blocked for 20 min to prevent unspecific antibody binding using Fc-block (BioXCell). Single cell suspensions of isolated lymphocytes from the CNS, lymph nodes and spleen were stained for 30 min at 4 °C on the cell surface with antibodies against CD90.2 APC-Cy7 (53-2.1, rat monoclonal, 1:1000, eBioscience), CD4 PerCP (GK1.5, rat monoclonal, 1:200, Biolegend), CD44 FITC (IM7, rat monoclonal, 1:200, eBioscience), CD45 BV510 (30-F11, rat monoclonal, 1:200, Biolegend), CD11b PECy7 (M1/70, rat monoclonal, 1:1000, eBioscience), Ly6c V450 (AL-21, rat monoclonal, 1:100 BD Bioscience), Ly6c PerCP (HK1.4, rat monoclonal, 1:200 Biolegend), Ly6G PE (1A8, rat monoclonal, 1:2000 Biolegend).

Afterwards, where indicated, cells were fixed and permeabilized with Cytofix/Cytoperm (BD Bioscience) and stained for 30 min at 4 °C with intracellular antibodies. To specifically gate on MOG-specific cells, cells were stained with anti-CD154 (CD40L) APC (MR1, hamster monoclonal, 1:200, Biolegend) and cytokine production was assessed by staining with IL-17A V450 (eBio17B7, rat monoclonal, 1:300, eBioscience), IFN-y PECy7 (XMG1.2, rat monoclonal, 1:400, eBioscienc) and GM-CSF PE (MP1-22E9, rat monoclonal, 1:200, eBioscience).

For EC staining, single cell suspension of isolated CNS cells were stained for 30 min at 4 °C for the following surface markers: CD31 PE, CD31 APC or CD31 FITC (all MEC 13.3, rat monoclonal, 1:100, BD), Icam-1 APC (YN1/1.7.4, rat monoclonal, 1:300, Biolegend), Vcam-1 PECy7 (MVCAM.A, rat monoclonal, 1:300, Biolegend), IL-1R1 PE (JAMA-147, rat monoclonal, 1:200, Biolegend). For intracellular staining, CNS cells were fixed and permeabilized using Cytofix/Cytoperm kit and stained for 30 min at 4 °C against Darc PE (Met1-Pro61, goat monoclonal, 1:30, R&D) and HO-1 (HO-1-1, mouse monoclonal, 1:300, Enzolifescience). Afterwards, samples were washed with PBS/FCS and incubated for 30 min at 4 °C with secondary antibody IgG Alexa488 (goat anti mouse, 1:200, Jackson) for HO-1 staining. Stained cells were acquired with a FACSCanto II cytometer (BD Biosciences) using FACS Diva software (BD Biosciences). Flow cytometry data were analyzed with FlowJo software version 9 or higher (TreeStar). For all analysis, doublets (FSC and SSC properties) and dead cells (dye inclusion) were excluded.

### Use of the RiboTag method to isolate RNA

BBB-EC ribosomes from spinal cord of EAE immunized mice were isolated using RiboTag technology as described previously [66]. Briefly, spinal cord tissue was removed, weighed and homogenized in homogenization buffer (200 U/ml RNasin, Promega; 1 mg/ml heparin, Sigma-Aldrich; 100 μg/ml cycloheximide, Sigma-Aldrich; protease inhibitor mixture, Sigma-Aldrich; 1 mM Dithiothreitol, Boehringer Mannheim GmbH), using 2–3% weight per volume for immune-precipitation (IP). The homogenate was centrifuged at 1000×*g* for 10 min at 4 °C and the supernatant was mixed with HA tag antibody (C29F4, rabbit monoclonal, 2 μg/ml, cell signaling) and rotated for 4 h at 4 °C. Afterwards, the spinal cord homogenate was mixed with 200 μl Protein G magnetic beads (Invitrogen) overnight at 4 °C. The following day, the samples were placed into a magnetic rack and supernatant was removed before washing the beads three times for 10 min in high salt buffer (50 mM Tris, pH 7.5, 300 mM KCl, 12 mM MgCl2, 10% NP-40, 1 mM dithiothreitol and 100 μg/ml cycloheximide which was supplied by Sigma-Aldrich). Immediately after removal of the final high salt wash buffer, lysis buffer (Qiagen) was added and samples were vortexed. Magnetic beads were removed by placing samples again into a magnetic rack and mRNA was extracted.

### RNA isolation

mRNA was prepared using the Rneasy^®^ Plus Micro Kit from Qiagen according to the manufacturer’s guidelines. The concentration of nucleic acids was determined by measuring the absorption at 260 nm and 280 nm using NanoDrop™ (Thermo Fisher Scientific). Equal amounts of RNA for all samples were used for following assays.

### Quantitative real-time PCR

cDNA was synthesized using 200–1000 ng of total RNA with the superscript II reverse transcriptase (Invitrogen) and subsequently used for qPCR, which was performed with the StepOnePlus™ Real-Time PCR System (Life Technologies) using SYBR Green (Promega). Fold enrichment was calculated using the Delta–Delta CT method normalized to hypoxanthin-guanin-phosphoribosyltransferase (*HPRT*) as house-keeping reference. Primer for *HPRT*, *Hmox1*, *Icam1*, *Vcam1* and *IL-1R1* were purchased from Qiagen. *Ackr1* primer (forward: 5′-CTT CAC CTT GGG ACT CAG TGT-3′; reverse: 5′-GAC TGG CAG CCC TAA GAG G-3′) were self-designed using Primer Express 3.0 software and were synthesized by Metabion.

### Next-generation sequencing (NGS)

RNA of RiboTag isolated tissue was quantified with a Qubit 2.0 fluorometer (Invitrogen) and the quality was assessed on a Bioanalyzer 2100 (Agilent) using an RNA 6000 Nano chip (Agilent). Samples with an RNA integrity number (RIN) of > 8 were used for library preparation. Barcoded mRNA-seq cDNA libraries were prepared from 200 ng of total RNA using NEBNext^®^ Poly(A) mRNA Magnetic Isolation Module and NEBNext^®^ Ultra™ RNA Library Prep Kit for Illumina^®^ according to manufacturer’s guidelines. Quantity was assessed using Invitrogen’s Qubit HS DNA assay kit and library size was determined using Agilent’s 2100 Bioanalyzer HS DNA assay. Barcoded RNA-Seq libraries were onboard clustered using HiSeq^®^ Rapid SR Cluster Kit v2 using 8 pM and 59bps were sequenced on the Illumina HiSeq2500 using HiSeq^®^ Rapid SBS Kit v2 (59 Cycle). The raw output data of the HiSeq^®^ were preprocessed according to the Illumina standard protocol.

### NGS data analysis

Quality control on the sequencing data was performed with the FastQC tool (version 0.11.2, https://www.bioinformatics.babraham.ac.uk/projects/fastqc/). RNA sequencing reads were aligned to the ENSEMBL Mus_musculus.GRCm38 reference genome. The corresponding annotation (ENSEMBL v76) was also retrieved from ENSEMBL FTP website. The STAR aligner (version 2.4.0j) was used to perform mapping to the reference genome. Alignments were processed with the featureCounts function of the Rsubread package [41], using the annotation file also used for supporting the alignment. Differential expression analysis was performed with DESeq2 package (version 1.22.1) [48], setting the false discovery rate (FDR) cutoff to 0.1. Accurate estimation of the effect sizes (in terms of log fold change) is performed using the apeglm shrinkage estimator (version 1.4.1) [88]. Gene expression profiles were plotted as heatmaps (color-coded *z*-scores for the expression values, after regularized logarithm transformation) with the R programming language (https://www.R-project.org/) and the heatmap package (version 1.0.12).

Principal component analysis was performed using the pcaExplorer package version2.8.0 [51]. To highlight the differences of the expression values between the two groups, MA-Plots were generated with the R programming language. Further analyses included Gene Ontology pathway enrichment of DEG using topGO [5] (v2.34.0) and goseq (v1.34.0) [86] (with all expressed genes set as background) were performed using the ideal package (version 1.6.0) [52].

### Evans Blue assay

2% Evans Blue (Sigma-Aldrich) solution was intraperitoneally injected at onset of EAE disease. After 6 h, perfused spinal cords were weighed and homogenized in 1 ml 50% trichloroacetic acid (Sigma-Aldrich) and centrifuged for 10 min at 1000×*g* and 4 °C. Evans Blue concentration in the supernatant was measured on a plate reader (Infinite M200, Tecan Life Sciences) (excitation at 620; emission at 680) and quantified according to a freshly prepared standard curve.

### FITC dextran assay

Mice were intravenously injected with 5 mg 20 kDa FITC-Dextran (Sigma-Aldrich) in 0.9% NaCl at day 10 after EAE induction (before clinical onset). After 15 min, perfused spinal cords were weighed and homogenized in 1 ml 60% trichloroacetic acid using a Dounce homogenizer (Kimble Chase Life Science and Research Products, LLC). Samples were centrifuged at 1000×*g* for 10 min. Fluorescence was measured in the supernatant on a fluorescence plate reader (Tecan Life Technologies) with 470 nm excitation and 520 nm emission, and quantified according to standard curve per mg of tissue.

### Immunofluorescence

For fluorescence microscopy, mice were deeply anesthetized with isoflurane and perfused with 0.9% NaCl followed by 30 ml of 4% paraformaldehyde (PFA) (Sigma-Aldrich) in PBS. Spinal cord was removed and post-fixed overnight in 4% PFA followed by 3 days incubation in 30% sucrose (Sigma-Aldrich). Sections (10 μm) were blocked using 1/1000 immunoblock (Roth) or 5% normal goat serum (NGS) respectively, and 0.5% Triton-X100 (Sigma-Aldrich) in PBS for 1 h at RT. The following primary antibodies were incubated at 4 °C overnight: CD31 (rabbit polyclonal, 1:100, Abcam), HA tag (C29F4, rabbit monoclonal, 1:800, Cell signaling), IgG biotinylated (rabbit polyclonal, 1:500, Vector), CD3 (HH3E, rat monoclonal, 1:100, Dianova), Laminin (rat polyclonal, 1:200, DAKO), CD11b (5C6, rabbit monoclonal, 1:200, Bio Rad formerly Serotec), Iba-1(rabbit polyclonal, 1:200, Synaptic Systems). After washing, sections were stained for 1 h at RT in the dark with the following secondary antibodies: anti-goat IgG Alexa488 (1:250, Invitrogen), anti-rat IgG Alexa 568 (1:250, Invitrogen), anti-mouse IgG Alexa488 (1:200, Jackson) or using streptavidin Cy3 (1:200, Jackson) for biotinylated antibodies. For Myelin staining, sections were stained for 20 min at RT with FluoroMyelin™ Red Fluorescent Myelin Stain (Thermo Fisher).

Afterwards, slides were mounted in Vectashield containing DAPI (Vector). Images were acquired with Olympus Fluoview 100 or Zeiss sp8 and analyzed in Fiji.

### In vitro culture of bEnd.3 cell line

The bEnd.3 cells (BEND3) (ATCC^®^ CRL-2299™, RRID:CVCL_0170) were cultured in DMEM with 10% FCS, according to the standard protocol and maintained in 37 °C and 10% CO_2_. Cells were split every 2–3 days and used at approximately 90% confluency.

### Isolation and culture of primary endothelial cells

Cortices from 4–10-week-old WT mice were dissected. Outer vessels and meninges were removed using a dry cotton swap. Tissue was pooled and homogenized in a Dounce homogenizer (Kimble Chase Life Science and Research Products, LLC) in HBSS with 0.1% BSA (Sigma-Aldrich) and 1 mM HEPES (Gibco). The homogenate was mixed with 30% dextran (Sigma-Aldrich) in HBSS, BSA, HEPES and centrifuged at 3000×*g* for 25 min at 10 °C. The pellet was collected and dextran solution was again centrifuged as before. After second centrifugation, the first and second pellet were pooled and washed with homogenization buffer (HBSS with 0.1% BSA and 1 mM HEPES) and filtered through a 70 μm cell strainer. Capillary enriched filtrate was centrifuged at 1000×*g* for 10 min at 10 °C and subsequently digested in 2 mg/ml collagenase/dispase (Roche), 10 μg DNase I (Roche) and 147 μg/ml Na-Tosyl-l-lysyl-chloromethane hydrochloride (Sigma-Aldrich) in HBSS for 30 min at 37 °C. Afterwards, vessels were filtered and washed through a 20 μm nylon filter and seeded on 48-well culture dishes coated with Matrigel (ECM Gel from Engelbreth-Holm-Swarm murine sarcoma, supplied by Sigma Aldrich). Culture medium was supplemented with 20% FCS, 2% non-essential amino acids and 5 μ/ml gentamycin. 1 ng/ml human fibroblast growth factor (FGF) (Sigma-Aldrich) and 4 μg/ml puromycin (Gibco) were added for the first 48 h of culture. Prior to each medium change (every second day), medium was supplemented with fresh FGF and cells were kept in culture for 6 days.

### In vitro endothelial cell stimulation assay

Cultured cells were incubated with the indicated concentration of hemin (Sigma-Aldrich) to induce HO-1 expression. Simultaneously, cells were stimulated with different concentration of IL-1β (Sinobiological), as indicated, for 16 h. All stimulation experiments were performed in DMEM containing 2% FCS and 25 mM HEPES at 37 °C and 10% CO_2_. For downstream analysis, cells were either lysed for RNA isolation according to the manufacturer’s protocol of the RNeasy^®^ Plus Micro Kit (Qiagen) or trypsinized to proceed with flow cytometry staining.

### Statistical analysis

Statistical analysis was performed with Prism (Graph Pad, Version 5.0b). Differences were evaluated by unpaired, two-tailed Students *t* test, by one-way ANOVA followed by tests with Bonferroni correction, or two-way ANOVA, as indicated. All values are represented as mean ± SEM and *p* values were considering significance threshold *p* < 0.05*; *p* < 0.01**; and *p* < 0.001***.

### Data availability

The sequencing datasets generated during the current study have been deposited in the Gene Expression Omnibus (GEO) archive, and are available under the accession number GSE146378.

## Results

### IL-1R1 signaling in BBB-ECs is critically involved in the development of EAE

To assess IL-1R1 expression on different CNS-resident cell types, we induced active EAE in wild-type mice and isolated CNS tissue at different time points after immunization. In accordance with published data [39], we observed that 15% of BBB-ECs (defined as living CD45^−^CD11b^−^CD31^+^) expressed the IL-1R1 as early as 3 days post-immunization (Supplementary Fig. 1a–c, online resource) as a result of innate activation caused by the CFA injection (Supplementary Fig. 2, online resource). In contrast, neither microglia (defined as living CD45^+^CD11b^int^) nor astrocytes (defined as living CD45^−^CD11b^−^GFAP^+^) were positive for IL-1R1 expression, regardless of the EAE stage (Supplementary Fig. 1b, online resource). Together, these data demonstrate that IL-1R1 expression in the CNS is rather restricted to BBB-ECs.

**Fig. 1:**
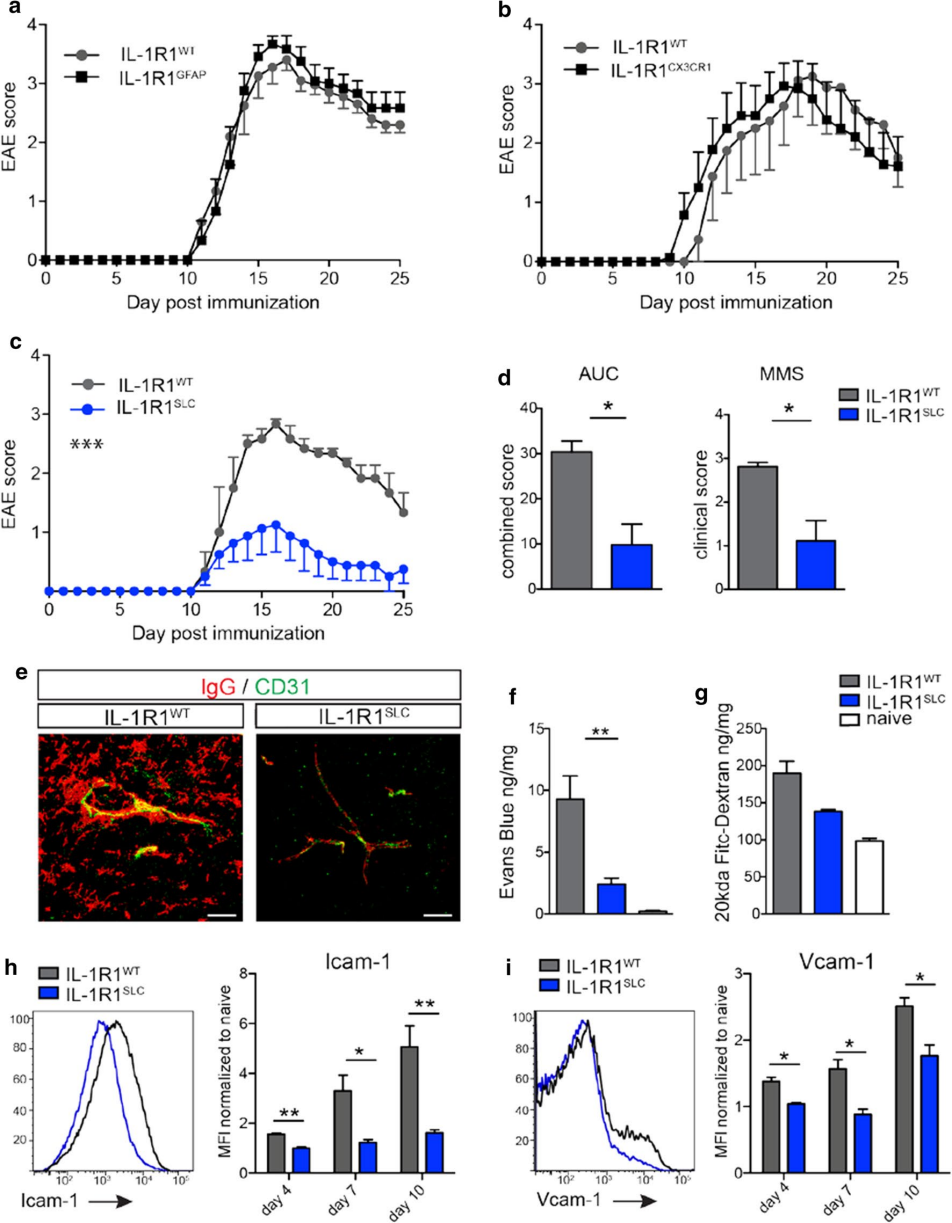
BBB-EC IL-1 signaling drives EAE and promotes Icam-1 and Vcam-1 expression. Disease course of IL-1R1^WT^ and IL-1R1^GFAP^ (**a**) or IL-1R1^WT^ and IL-1R1^CX3CR1^ (**b**) mice. Data in **a** and **b** is representative for three individual experiments with at least *n* = 6 per group. Disease course (**c**) and area under the curve (AUC) and mean maximum score (MMS) (**d**) of IL-1R1^SLC^ and IL-1R1^WT^ mice. Data in **c** and **d** is representative for three individual experiments with at least *n* = 5 per group. **e** Representative 3D-images of immunohistochemistry staining from spinal cord sections at EAE onset stained for IgG (red) and CD31 (green). Indicated scale bar = 20 μm. **f** Extravasation of Evans Blue into spinal cords at EAE onset. Quantification was performed 6 h after intravenous injection of the dye. Data in **f** is representative for three individual experiments with at least *n* = 5 per group. **g** FITC-dextran (20 kDa) was applied intravenously into mice at day 10 post-immunization and tracer accumulation in spinal cord tissue was quantified 15 min later. Data in **g** is representative for three individual experiments with at least *n* = 4 per group. Flow cytometry analysis of CNS tissue at days 4, 7 and 10 post-immunization showing mean fluorescence intensity (MFI) of Icam-1 (**h**) and Vcam-1 (**i**) on BBB-ECs (gated as CD45^−^CD11b^−^CD31^+^Ly6c^+^ living single cells) normalized to naïve (unimmunized) mice. Data in **h** and **i** is representative for three individual experiments with at least *n* = 4 per group. Data in **a–d** and **f–i** is shown as mean ± SEM and analyzed using two-tailed unpaired Student’s *t* test (**d**,** h**, **i**) or two-way ANOVA (**a**–**c**,** f**–**g**). *p < 0.05, **p < 0.01, ***p < 0.001

Next, we aimed to investigate the cell type-specific role of IL-1 signaling in CNS-resident cells for EAE pathogenesis and progression. For that, we used a mouse line, in which exon 5 of the *Il1r1* gene is flanked by loxP sites, thereby enabling Cre-mediated recombination and subsequent IL-1R1 deletion, rendering the targeted cell type refractory to IL-1α and IL-1β signaling [2]. To delete IL-1R1 in astrocytes, we crossed these mice to the *Gfap-Cre* mice, which have previously been used successfully for targeted mutation approaches in astrocytes [10, 83]. For IL-1R1 deletion in microglia, we used the *Cx*_*3*_*cr1-Cre*^*ERT2*^ mice, where tamoxifen (TAM) injection induces Cre expression specifically in microglia and CNS border-associated macrophages (BAMs), which are targeted due to their long living and self-maintaining capacity [29, 59, 85]. In agreement with the lack of IL-1R1 expression in these cell types (Supplementary Fig. 1, online resource), mice devoid of IL-1 signaling in astrocytes (IL-1R1^GFAP^) or in microglia and BAMs (IL-1R1^CX3CR1^) showed unaltered susceptibility to EAE induction as compared to control wild-type animals (Fig. 1a, b). Together, these data suggest that IL-1 signaling in astrocytes and microglia is not important for the development of autoimmune neuroinflammation in the EAE model.

To address the role of IL-1 signaling in BBB-ECs during EAE pathogenesis, we crossed the conditional *Il1r1*^*fl/fl*^ mice to the TAM-inducible BBB-EC-specific *Slco1c1-Cre*^*ERT2*^ mice [65], allowing for tissue-specific deletion of IL-1R1, hereafter termed IL-1R1^SLC^ mice. To induce Cre-mediated IL-1R1 deficiency, these mice received TAM injection at 6 weeks of age and were subjected to active EAE immunization 4 weeks later. Using flow cytometry analysis, we could show that IL-1R1 was indeed efficiently deleted in ECs isolated from brain and spinal cord (Supplementary Fig. 1d, online resource). Of note, the TAM injection itself neither altered the IL-1R1 expression in ECs, nor did it impact the production of IL-1β by peripheral myeloid cells (Supplementary Fig. 3, online resource). Importantly, deletion of IL-1 signaling in ECs largely protected mice from EAE induction (Fig. 1c), as evidenced by significantly reduced overall disease severity and mean maximum scores (Fig. 1d).

As EAE severity positively correlates with the disruption of BBB integrity, we stained spinal cord sections for serum IgG to test BBB permeability. As can be seen in Fig. 1e, IL-1R1^SLC^ mice showed much less passive diffusion of IgG into the inflamed spinal cord tissue compared to control animals at the onset of EAE disease. Similarly, these mice were largely protected from Evans Blue leakage into CNS parenchyma (Fig. 1f), confirming that IL-1R1^SLC^ mice show less BBB damage at EAE onset. However, we did not detect a significant difference in the translocation of the smaller 20 kDa FITC-dextran, when injected intravenously (i.v.) at day 10 post-immunization (Fig. 1g), indicating that the discrete focal disturbances of BBB integrity before clinical onset of the disease cannot be sufficiently reflected by the gross measurement of indicator molecule infiltration.

Importantly, flow cytometric analysis of ECs (CD45^−^CD11b^−^, CD31^+^Ly6c^+^) isolated from the CNS of IL-1R1^SLC^ mice revealed reduced expression of Icam-1 and Vcam-1 during the preclinical EAE phase (days 4, 7 and 10 post-immunization) compared to control BBB-ECs (Fig. 1h, i). Together, these data demonstrate that IL-1 signaling, specifically in ECs, promotes BBB permeability once clinical EAE is initiated. Furthermore, IL-1 signaling on BBB-ECs controls the upregulation of prototypical genes associated with EC activation, including Icam-1 and Vcam-1. Our findings, therefore, suggest a direct involvement of IL-1 signaling in ECs in the recruitment of peripheral immune cells into the CNS parenchyma.

### The endothelial response to IL-1 drives leukocyte infiltration

**Fig. 2:**
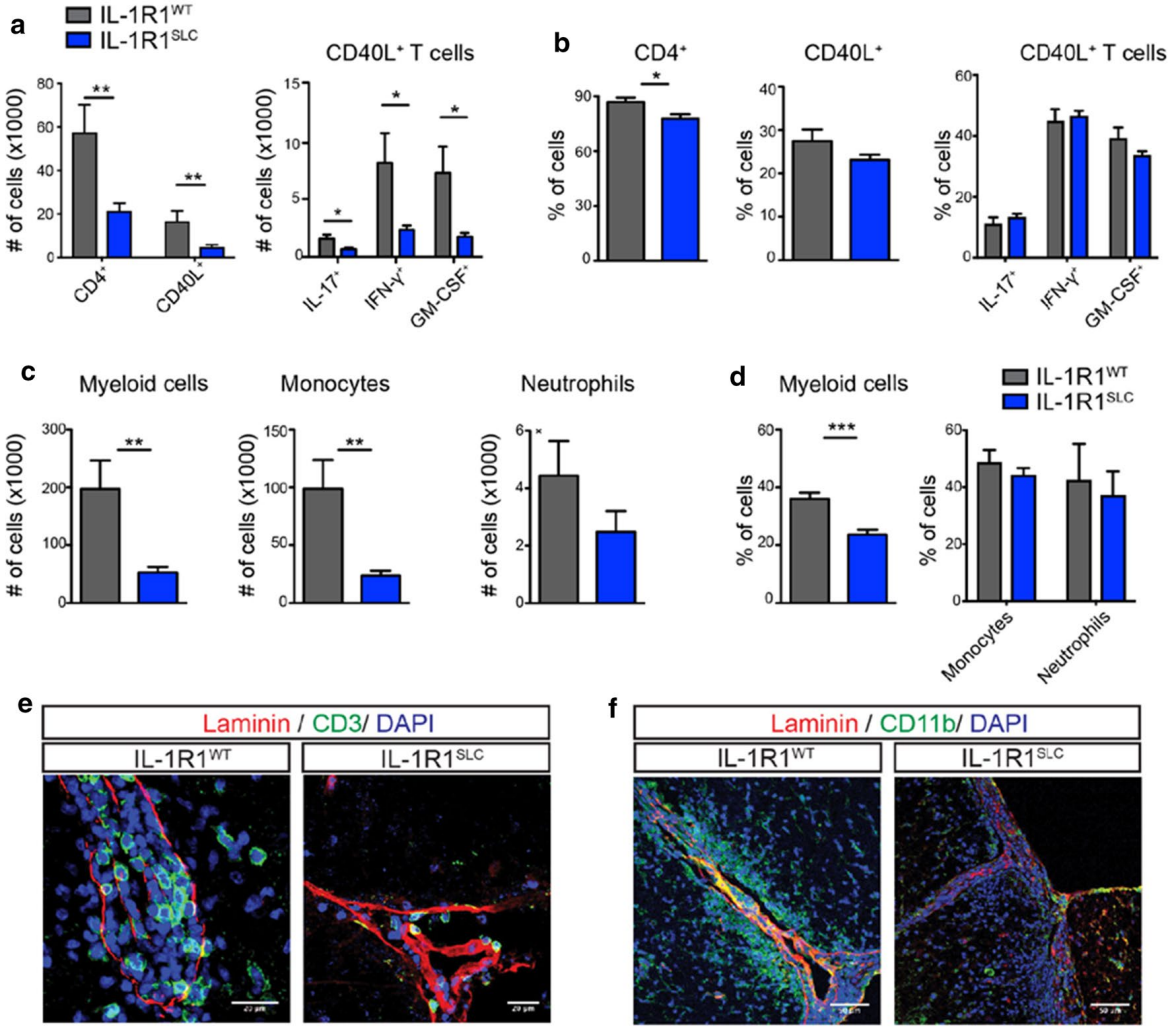
BBB-EC IL-1 signaling promotes leukocyte migration. **a**, **b** Spinal cords of IL-1R1^SLC^ and control mice at peak of EAE disease were isolated and single cell suspensions were subjected to MOG antigen recall assay. Flow cytometry analysis was quantified and shows the absolute cell number (**a**) and frequency (**b**) of the indicated T cell populations. Note that CD40L^+^ cells are considered to be specific for the MOG antigen. **c**, **d** Single cell suspensions from spinal cords of peak EAE mice were analyzed by flow cytometry for CNS-infiltrating myeloid cells. The quantification of this analysis shows the absolute cell number (**c**) and frequency (**d**) of living CD45^+^CD11b^high^ myeloid cells, further gated on Ly6c^high^ monocytes and Ly6c^+^Ly6G^+^ neutrophils. Data in **a**–**d** is representative for at least three individual experiments with a minimum of *n* = 5 per group. Data is shown as mean ± SEM and analyzed using two-tailed unpaired Student’s *t* test. *p < 0.05, **p < 0.01, ***p < 0.001. Immunofluorescence analysis of spinal cord tissue at EAE onset showing CD3^+^ T cells (**e**) and CD11b^+^ myeloid cells (**f**) infiltrating the CNS parenchyma. Spinal cord sections were stained for CD3 (green, in **e,** scale bar = 20 μm), CD11b (green, in **f**, scale bar = 50 μm) and DAPI (blue) together with pan-laminin staining (red)

Next, we investigated whether the resistance to EAE observed in IL-1R1^SLC^ mice was associated with reduced CNS leukocyte infiltration. For that, we analyzed the presence of MOG-specific T cells in the brain and spinal cord of IL-1R1^SLC^ and control animals at peak of EAE. To specifically characterize MOG-specific T cells, we re-activated cellular infiltrates isolated from the CNS for 6 h in vitro with MOG peptide and analyzed the expression of CD40L on T cells as marker of recent activation [25]. We noted a significant reduction of total antigen-specific CD4^+^ T cells producing IL-17A, IFN-γ and GM-CSF entering the CNS of IL-1R1^SLC^ mice (Fig. 2a and Supplementary Fig. 5, online resource). Importantly, we found no bias in the proportions of the different T cell subsets (Fig. 2b and Supplementary Fig. 5, online resource), indicating that IL-1 signaling in BBB-ECs does not regulate the entry of specific T helper sub-populations to the CNS during EAE. Of note, relative and absolute numbers of T cell subsets infiltrating the brain parenchyma revealed the same patterns as seen for the spinal cord (Supplementary Fig. 4a, Supplementary Fig. 4c, d and Supplementary Fig. 5, online resource).

Importantly, before clinical onset of the disease (day 9), absolute numbers and the composition of T helper cell subsets in the lymph nodes and spleen of IL-1R1^SLC^ mice were very similar to that of control mice (Supplementary Fig. 4g and 4h, online resource). This suggests that the reduced infiltration of encephalitogenic T cells in the spinal cord of IL-1R1^SLC^ mice was likely due to IL-1-driven changes at the level of the BBB rather than general failure in differentiation and expansion of effector CD4 T cell subsets.

Next, we assessed the proportions of infiltrating myeloid cells, previously shown to promote EAE development [22]. In agreement with the reduced numbers of lymphocytes, we found a significant reduction in frequency and numbers of inflammatory monocytes (CD45^+^CD11b^high^Ly6c^high^) in the spinal cord of IL-1R1^SLC^ mice. In contrast, the number of CNS-infiltrating neutrophils (CD45^+^CD11b^high^Ly6g^+^Ly6c^+^) was not altered (Fig. 2c, d). Similar findings were observed also in the brain of IL-1R1^SLC^ mice (Supplementary Fig. 4b and Supplementary Fig. 4e–f, online resource). Interestingly, although the lack of endothelial IL-1 signaling significantly altered the transmigration of myeloid cells, their capacity to produce IL-1β was unchanged (Supplementary Fig. 6, online resource).

As we found reduced numbers of infiltrating cells in the inflamed CNS of IL-1R1^SLC^ mice, we set to determine whether there were also differences in their localization. Immunohistochemistry staining for CD3^+^ T cells and CD11b^+^ myeloid cells in conjugation with pan-laminin visualization was performed to assess cell frequencies within the perivascular space and the surrounding CNS parenchyma. In line with our flow cytometric quantification, we found significantly fewer T cells as well as myeloid cells within perivascular cuffs, alongside strongly reduced numbers of transmigrated cells in the spinal cord parenchyma in IL-1R1^SLC^ compared to control mice (Fig. 2e, f). Moreover, IL-1R1^SLC^ mice also showed markedly fewer activated Iba1^+^ microglia as well as significantly less demyelination at sites of immune cell infiltration (Supplementary Fig. 7 online resource).

### Endothelial IL-1 signaling represses the expression of HO-1

**Fig. 3:**
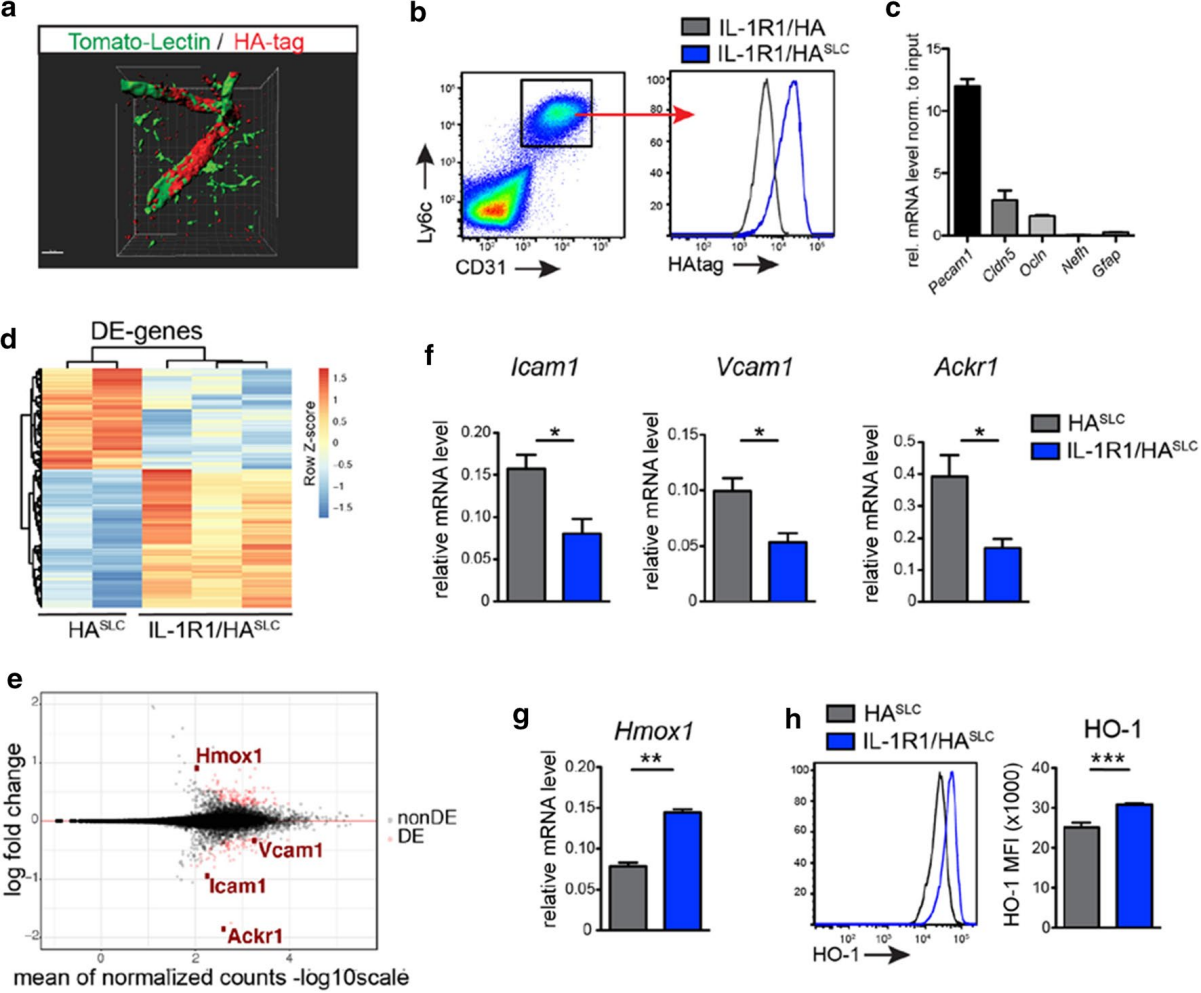
IL-1R1-deficient BBB-ECs show increased HO-1 expression before onset of EAE. **a** 3D reconstruction of confocal images from spinal cord sections of IL-1R1/HA^SLC^ mice stained for HA-tag (red) and Lectin (green). Images were generated with Imaris software. **b** Flow cytometry analysis of HA-tag expression by BBB-ECs (gated as CD45^−^CD11b^−^CD31^+^Ly6c^+^ living single cells) from mice of the indicated genotypes. **c** Quantification of the indicated genes by qRT**-**PCR of immunoprecipitated (IP) mRNA from spinal cord tissue of HA^SLC^ mice. Expression levels are shown as log fold change normalized to input fraction. Data is representative for two individual experiments with at least n = 3 per group and shown as mean ± SEM. **d** Heat map of RNA sequencing data comparing IPs obtained from spinal cords of HA^SLC^ (*n* = 2) and IL-1R1/HA^SLC^ (*n* = 3) mice at day 10 after EAE induction. Each column represents one individual mouse. Color-coded *z*-scores for the regularized logarithm (rlog) transformed expression values are displayed. **e** MA-plot displaying expression change (in log2 scale) versus mean expression values. Each dot represents one gene with differentially expressed (DE) genes in red. **f**, **g** qRT-PCR validation showing top downregulated genes *Icam1*, *Vcam1*, *Ackr1* (**f**) and the top upregulated gene *Hmox1* (**g**), as obtained from the RNA sequencing analysis. Data in **f** and **g** is representative for two individual experiments with at least *n* = 3 per group**. h** Flow cytometry analysis of BBB-ECs showing mean fluorescence intensity (MFI) of HO-1. Data in **h** is representative for two individual experiments with at least *n* = 4 per group. Data in **f–h** is shown as mean ± SEM and analyzed using two-tailed unpaired Student’s *t* test. *p < 0.05, **p < 0.01, ***p < 0.001

After establishing a crucial link between IL-1 signaling and BBB-ECs for EAE pathogenesis, we aimed to investigate IL-1-mediated changes in ECs at a transcriptomic level. For this purpose, we made use of the RiboTag technology, which allows for the isolation of actively transcribed (ribosome-engaged) mRNA of specific cell types in a Cre-dependent manner [31, 66]. We crossed the RiboTag mouse to the BBB-specific *Slco1c1-Cre*^*ERT2*^ mouse, resulting in HA^SLC^ mice, and injected them with TAM to enable HA tag expression. To verify the correct targeting of ECs, we analyzed HA tag expression by immunohistochemistry and flow cytometry of CNS tissue. As can be seen in Fig. 3a, b, both methods verified the expression of the tagged ribosomes in ECs. We next isolated tagged ribosomes (including the associated mRNA) by immunoprecipitation (IP) from spinal cord lysates of HA^SLC^ mice. Using qRT-PCR, we could show the enrichment for EC-associated genes, including *Pecam1*, *Claudin5* and *Occludin1* and verify the EC-specific expression of HA-tagged ribosomes (Fig. 3c).

To interrogate how IL-1 signaling on BBB-ECs drives the pathogenesis of EAE and to identify the responsible target genes, we opted to compare the gene expression signature of BBB-ECs isolated from the spinal cords of IL-1R1^SLC^ and control mice at disease onset. Therefore, we crossed the RiboTag to the IL-1R1^SLC^ mice, resulting in IL-1R1/HA^SLC^ mice, whereas HA^SLC^ mice served as control animals. We isolated RiboTag-associated mRNA at day 10 after disease induction and performed high-throughput RNA sequencing. As can be seen in Fig. 3d, e, the deletion of IL-1R1 led to major transcriptional changes in ECs following EAE induction. qRT-PCR confirmation of the sequencing data showed the expression of *Icam1* (log2 fold-change = − 0.94), *Vcam1* (log2 fold-change = − 0.33) and *Ackr1* (log2 fold-change = − 1.8) being lower in the ECs lacking IL-1 signaling (Fig. 3f). These genes, coding for Icam-1, Vcam-1 and the atypical chemokine receptor 1 (Ackr1), also called Duffy antigen receptor for chemokines (Darc), were previously shown to promote EAE development by modulating transmigration of immune cells into CNS tissue [1, 27, 56, 73]. On the other hand, several genes were significantly upregulated in IL-1R1/HA^SLC^ mice at EAE onset, including *Hmox1*, coding HO-1, the most prominent candidate (log2 fold-change = 0.9), which was further confirmed by qPCR (Fig. 3g) and flow cytometry (Fig. 3h, Supplementary Fig. 8, online resource). Thus, the RNA and protein analysis revealed that IL-1 signaling in BBB-ECs leads to upregulation of genes that were reported to promote EAE pathogenesis, and at the same time causes downregulation of HO-1, which was previously shown to suppress autoimmune processes [18].

### Endothelial HO-1 activity ameliorates EAE disease

**Fig. 4:**
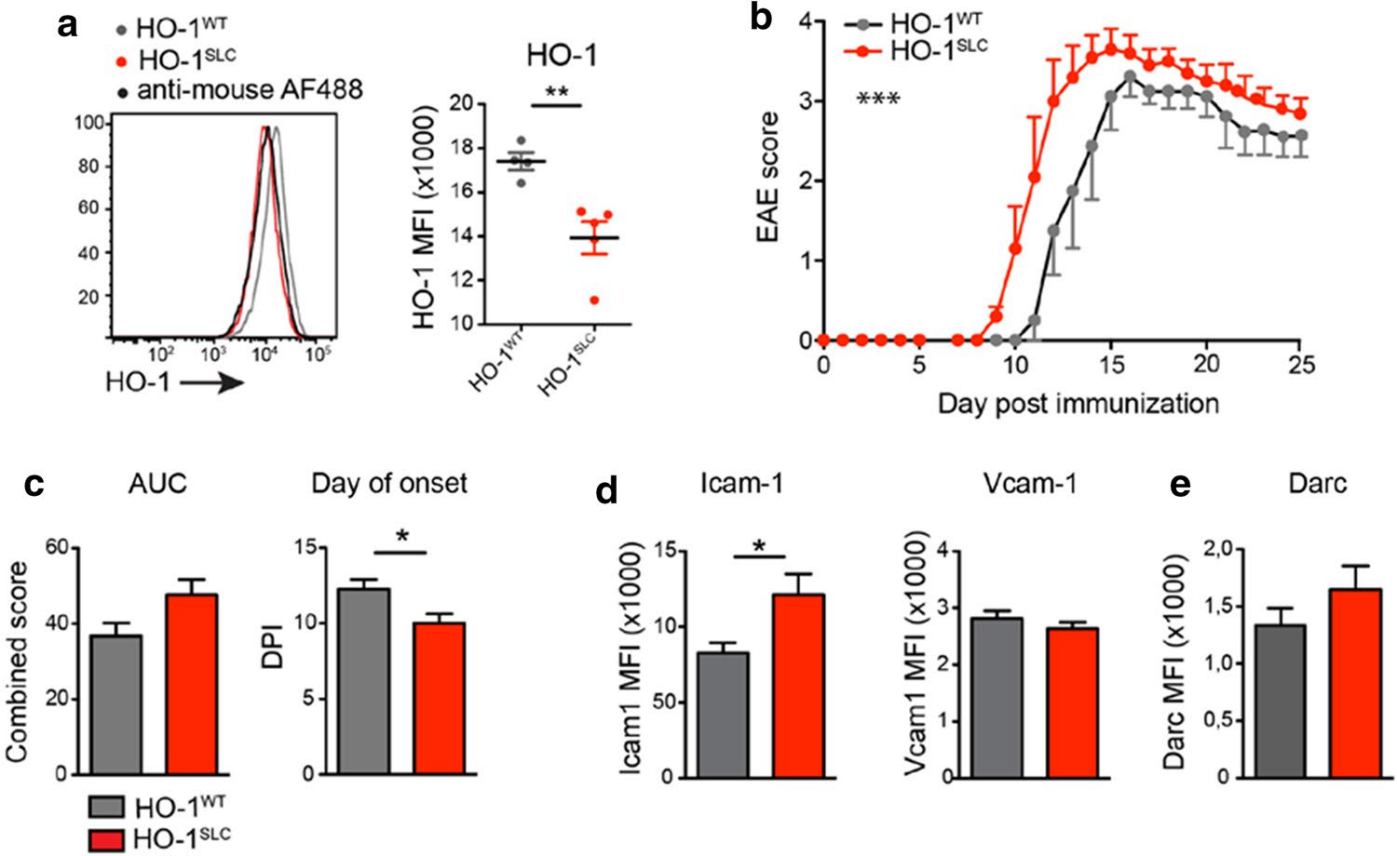
Increased EAE severity in mice lacking HO-1 expression in BBB-ECs. **a** Flow cytometry analysis from CNS tissue of the indicated mice afflicted with EAE, showing mean fluorescence intensity (MFI) of HO-1 in BBB-ECs (gated as CD45^−^CD11b^−^CD31^+^Ly6c^+^ living single cells) at day 10 post-immunization. Data are representative for two individual experiments with at least *n* = 4 per group. **b**, **c** The indicated mice were immunized for EAE induction and the disease course monitored (**b**) as well as the area under the curve and day of onset quantified (**c**). Data in **a**–**c** is representative for two individual experiments with at least *n* = 4 per group. **d**, **e** Quantification of flow cytometry analysis from CNS tissue showing mean fluorescence intensity (MFI) of Icam-1 and Vcam-1 (**d**) and Darc (**e**) expression in BBB-ECs at day 10 post-immunization. Data in **d** and **e** is representative for three individual experiments with at least *n* = 3 per group. Data in **a**–**e** is shown as mean ± SEM. Data in **a**,** c**–**e** was analyzed using two-tailed unpaired Student’s *t* test. Data in **b** was analyzed using two-way ANOVA. *p < 0.05, **p < 0.01, ***p < 0.001

The expression of HO-1 is regulated at the transcriptional level by a variety of stress and damage-response transcription factors [53], including the nuclear factor erythroid-2-related factor 2 (Nrf2) [4]. Heme catabolism by HO-1 occurs via a process cleaving the porphyrin ring of heme, thereby releasing equimolar amounts of iron, carbon monoxide (CO) and biliverdin, which is then converted to bilirubin by biliverdin reductase [30]. Heme catabolism by HO-1 in ECs acts in a cytoprotective manner via the production of CO [14], while repressing the expression of Icam-1 and Vcam-1 [72] via processes involving regulation of iron metabolism and interfering with NF-κB transcription factor activation [70]. Having established that CO generated via heme catabolism by HO-1 is protective against EAE [18], we reasoned that this may occur via a mechanism that involves the expression of HO-1 specifically in BBB-ECs, which is presumably impaired by IL-1R1 signaling in these cells. To test this hypothesis, we conditionally deleted *Hmox1* in BBB-ECs in HO-1^SLC^ mice. As HO-1 is predominantly increased in response to different forms of stress or damage [53], we verified gene deletion in HO-1^SLC^ mice, 10 days after EAE induction by flow cytometry (Fig. 4a). Compared to the control animals, HO-1^SLC^ mice showed an increase in EAE severity, accompanied by an earlier disease onset (Fig. 4b, c). Interestingly, flow cytometry analysis of ECs isolated from the CNS of HO-1^SLC^ mice also revealed a significant increase in Icam-1 expression 10 days post-immunization, whereas Vcam-1 levels remained unchanged, as compared to controls (Fig. 4d) and the expression levels of Darc were slightly increased in HO-1^SLC^ mice (Fig. 4e).

### Increased expression of HO-1 in BBB-ECs ameliorates EAE disease by suppressing IL-1β mediated gene expression

**Fig. 5:**
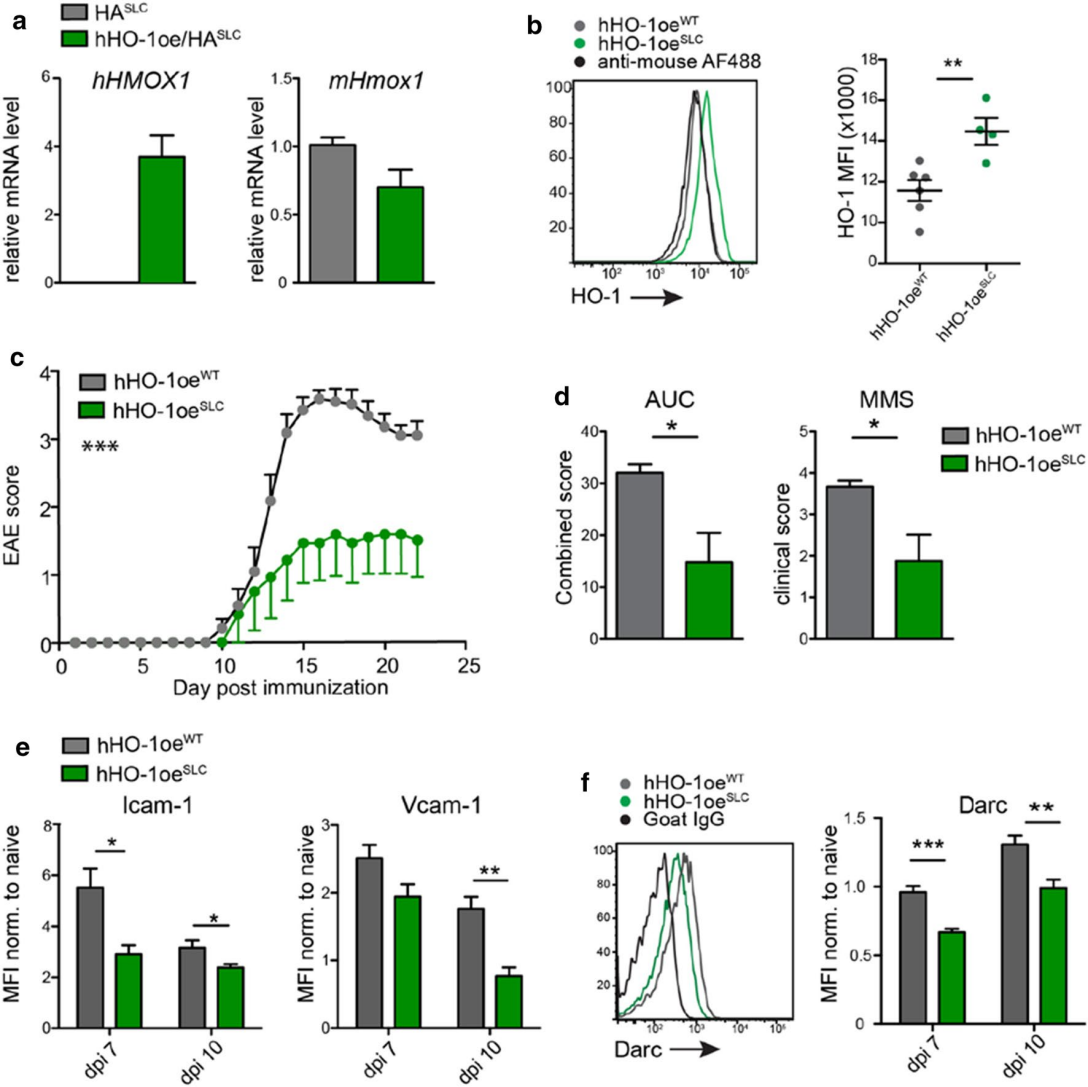
HO-1 overexpression by BBB-ECs reduces the expression of IL-1β target genes and EAE severity.** a** qRT-PCR for human *hHMOX1* (left) and mouse *mHmox1* (right) from RNA immunoprecipitated from spinal cords of the indicated mice (*n* = 3 per group). **b** Flow cytometry analysis of CNS tissue from EAE mice showing mean fluorescence intensity (MFI) of HO-1 expression by BBB-ECs (gated as CD45^−^CD11b^−^CD31^+^Ly6c^+^ living single cells) at day 10 post-immunization. Data is representative for two experiments with at least *n* = 4 per group. **c**, **d** The indicated mice were immunized for EAE induction and the disease course monitored (**c**) as well as the area under the curve and mean maximum score quantified (**d**). Data in **c** and **d** is representative for two individual experiments with *n* = 6 per group.** e**,** f** Flow cytometry analysis of CNS tissue showing mean fluorescence intensity (MFI) of Icam-1 and Vcam-1 (**e**) and Darc (**f**) expression by BBB-ECs at day 10 post-immunization. Data in **e** and **f** is representative for three individual experiments with *n* = 4 per group. Data in **a**–**f** is shown as mean ± SEM. Data in **a**,** b**, **d**–**f** was analyzed using two-tailed unpaired Student’s *t* test. Data in **c** was analyzed using two-way ANOVA. *p < 0.05, **p < 0.01, ***p < 0.001

Next, we tested whether HO-1 overexpression specifically in BBB-ECs would conversely affect the course of EAE disease. Therefore, we made use of mice that allow for the conditional overexpression of human *HMOX1* (*hHO-1oe*^*fl/fl*^ mice) [13], which we crossed to the *Slco1c1-Cre*^*ERT2*^ mouse line, resulting in the hHO-1oe^SLC^ mice. To facilitate gene expression analysis of ECs overexpressing the human HO-1, we crossed these mice also to the RiboTag mice mentioned above, allowing for isolation of mRNA from these ECs. Analysis of the HA-tagged precipitates from spinal cord tissue confirmed prominent expression of the human *HMOX1* (*hHMOX1*), whereas the intrinsic mouse *Hmox1* (*mHmox1*) expression remained unchanged (Fig. 5a). Furthermore, flow cytometric analysis at day seven after EAE induction revealed an increased expression of human and mouse HO-1 in BBB-ECs isolated from hHO-1oe^SLC^ mice as compared to control animals (Fig. 5b). Strikingly, in agreement with the increased EAE susceptibility of mice lacking HO-1 expression, mice overexpressing HO-1 in BBB-ECs were protected from EAE (Fig. 5c), as manifested by significantly reduced cumulative disease scores as well as mean maximum EAE severity in hHO-1oe^SLC^ mice compared to control animals (Fig. 5d). The overall reduced clinical disease was also associated with a significant reduction in CNS-infiltrating T cells as well as markedly reduced numbers of transmigrating myeloid cells (Supplementary Fig. 9, online resource).

Of note, BBB-ECs isolated from hHO-1oe^SLC^ mice showed significantly less Icam-1 and Darc expression already at preclinical (day 7 post-immunization) and early (day 10) disease stages (Fig. 5e, f), thereby complementing our previous data obtained with the IL-1R1^SLC^ EAE mice. In addition, Vcam-1 expression was significantly reduced at day 10 post-immunization in ECs of hHO-1oe^SLC^ mice (Fig. 5e). We conclude that HO-1 inhibits the expression of proinflammatory genes upregulated by IL-1 signaling and thereby limits leukocyte infiltration into the CNS during EAE pathogenesis.

### Crosstalk of IL-1 signaling and HO-1 activation in BBB-ECs

We then asked whether the suppression of IL-1-driven gene expression and induction of HO-1 expression in BBB-ECs of IL-1R1^SLC^ mice are independent events or if these two signals are integrated via molecular crosstalk. It has been demonstrated that the anti-inflammatory effects of HO-1 in ECs is exerted via inhibition of RelA phosphorylation at Ser276 [70], as RelA phosphor acceptor is required to induce several proinflammatory genes associated with EC activation, including Icam-1 and Vcam-1 [8]. We reasoned that HO-1 overexpression might repress EAE progression via a mechanism interfering with NF-κB activation downstream of the IL-1R1. HO-1 is transcriptionally inducible by a variety of agents, such as hemin [50], and its administration into EAE mice delayed the onset and reduced the severity of EAE efficiently [46]. To assess whether IL-1β can directly affect HO-1 expression under inflammatory conditions, we incubated CNS-derived endothelial cells (b.End3) with different concentrations of hemin to trigger HO-1 [72] expression in the presence or absence of IL-1β.

**Fig. 6:**
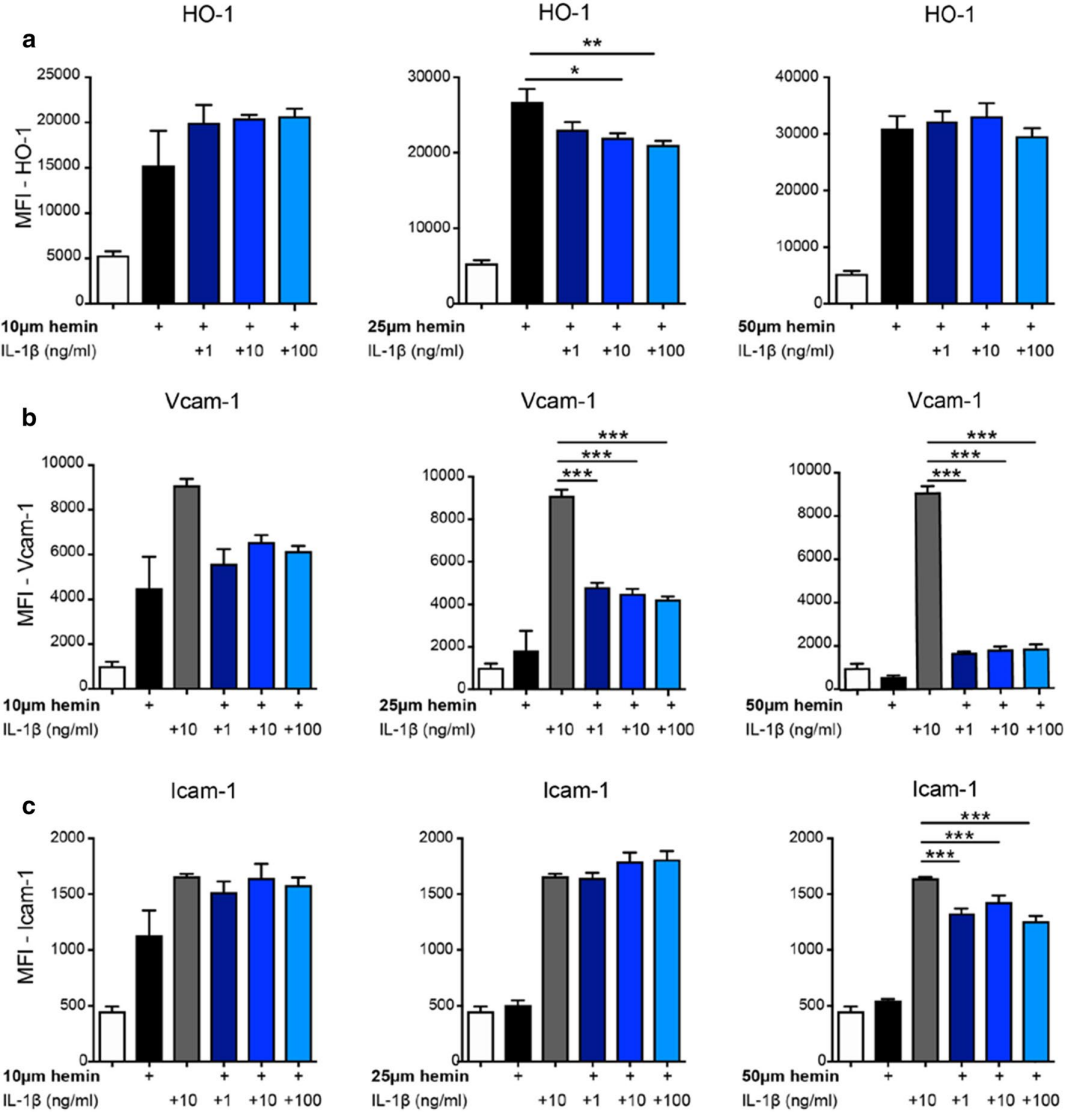
Molecular crosstalk of HO-1 and IL-1β signaling pathways. b.End3 cells were exposed to hemin, IL-1β or their combination at the indicated concentrations for 16 h. Flow cytometry analysis of stimulated cells was quantified and shows the mean fluorescence intensity (MFI) of HO-1 (**a**) Vcam-1 (**b**) and Icam-1 (**c**) expression. Data in **a**–**c** is representative for two individual experiments with at least *n* = 5 per group, and is shown as mean ± SEM and analyzed by one-way ANOVA with Bonferroni’s post hoc test. *p < 0.05, **p < 0.01, ***p < 0.001

After 16 h, cells were analyzed by flow cytometry, revealing that HO-1 expression was reduced after co-incubation with IL-1β (Fig. 6a). In addition, IL-1β-stimulated ECs showed a very efficient upregulation of Vcam-1 and Icam-1 protein levels (Fig. 6b, c). When we then co-incubated ECs with hemin and IL-1β, we found hemin to potently reduce the expression of Vcam-1 and Icam-1, regardless of the IL-1β concentration (Fig. 6b, c). Interestingly, Icam-1 was only reduced with higher doses of hemin (50 μM) (Fig. 6c). Since in our hands b.End3 cells did not show any Darc expression, we repeated in vitro experiments with primary mouse brain microvasculature ECs (pMBMECs) in the presence of 25 μM hemin together with 10 ng/ml of IL-1β. In accordance with the data obtained using the cell line, pMBMECs also upregulated HO-1 in response to hemin, while IL-1β suppressed this effect (Supplementary Fig. 10a, b, online resource). Importantly, in this setting, IL-1β potently induced not only *Vcam1* and *Icam1* expression, but also *Ackr1* and addition of 25 μM hemin was able to revert *Vcam1* and *Ackr1* expression (Supplementary Fig. 10c, online resource) in pMBMECs.

Together, our data demonstrate that IL-1 signaling and the HO-1 response in BBB-ECs regulate each other in a dose-dependent manner. This crosstalk might represent an important mechanism during inflammation and represents a promising target for therapeutic intervention.

## Discussion

The deletion of *Il1b* or *Il1r1* in mice leads to EAE resistance, emphasizing the importance of IL-1β as a critical mediator for the induction of CNS autoimmunity [39, 49, 68]. While it is well established that peripheral IL-1β acts primarily on T_H_ cells during the induction phase of EAE [61, 77], the cellular targets of this cytokine in the CNS are not well described. Although existing literature supports the notion that IL-1β may act directly on astrocytes and microglia [11, 58, 64], the response of these cells to IL-1β and the resulting consequences for EAE pathogenesis are still under debate. Using transgenic mouse lines in which *Il1r1* is deleted in specific CNS cellular subsets, we show that IL-1 signaling in astrocytes and microglia is dispensable for the pathogenesis of EAE. In contrast, *Il1r1* deletion specifically in BBB-ECs reduces EAE disease severity. Our study confirms that EC-IL-1 signaling plays a key role in mediating leukocyte migration across the BBB during neuroinflammation, which is a decisive factor for disease initiation [16, 17, 40, 55]. Importantly, we found that IL-1 signaling at the onset of EAE suppresses the expression of HO-1 in BBB-ECs and that HO-1 overexpression specifically in BBB-ECs reduces EAE severity, similar to what we observed in BBB-EC-specific *Il1r1* knockout mice. The protective effect of HO-1 is at least partially mediated by downregulation of IL-1β-driven gene expression, as illustrated for the adhesion molecules *Icam1* and *Vcam1* as well as the atypical chemokine receptor *Ackr1*, all of which are also downregulated in the absence of IL-1R1 in BBB-ECs during EAE. While reduced expression of Icam-1 and Vcam-1 partly accounts for the diminished leukocyte infiltration, downregulation of the atypical chemokine receptor 1 (Ackr1), also known as Duffy antigen receptor for chemokines (Darc), most likely contributes to EAE amelioration by preventing chemokine shuttling from the abluminal to the luminal surface of endothelial cells, thus counteracting the pro-migratory function of Darc during neutrophil infiltration [27, 56].

Our study is the first to demonstrate a protective role for BBB-EC-derived HO-1 in the development of CNS autoimmunity. It was previously shown that global *Hmox1* deletion in mice results in enhanced EAE severity, which is associated with the inhibition of MHCII expression by APCs and accumulation of CD4^+^ and CD8^+^ T cells within the CNS [18]. While the protective effect of HO-1 is driven at least partially through the end product of heme degradation, namely CO [18], this does not rule out that other products that emerge in this pathway, such as biliverdin or bilirubin, act in a similar manner. Indeed, biliverdin exerts potent immunoregulatory effects in T_H_ cells [84] while bilirubin, the product of biliverdin conversion by biliverdin reductase, reduces chronic and acute EAE disease in rats [47].

The role of HO-1 as an anti-inflammatory and anti-oxidative molecule has been studied also in other disease models. HO-1 supports BBB integrity in the context of cerebral malaria, an often lethal neuroinflammatory syndrome that develops in response to *Plasmodium* infection [62] and when expressed in spinal cord ECs it protects the BBB against oxidative injury and limits the infiltration of leukocytes into the CNS [43]. Other studies have demonstrated that HO-1 activation in cultured ECs inhibits the NF-κB-driven *Vcam1* transcription, which agrees with our in vivo and in vitro findings [70, 72].

The mechanism by which EC-IL-1 signaling inhibits HO-1 expression and vice versa, how HO-1 inhibits IL-1 signaling in ECs is not completely understood. Several studies have shown that activation of the transcription factor Nrf2 correlates with the suppression of NF-κB signaling [12, 34, 72]. Moreover, it was shown, that transcription driven by canonical NF-κB may regulate the Nrf2-mediated expression of the antioxidant response element (ARE) by different mechanisms, suggesting a crosstalk between the Nrf2/HO-1 and the IL-1 signaling pathways. The activity of Nrf2 is regulated by the repressor protein Keap1 [32]. Under conditions of oxidative stress, Keap1 loses its ability to repress Nrf2 activity, which leads to Nrf2 nuclear accumulation, inducing the expression of stress-preventing genes such as *HMOX1* [87]. Others have shown that the NF-κB p65/RelA subunit can physically associate with Keap1 to promote its translocation into the nucleus, resulting in the inactivation of Nrf2 [87]. The most well-established interplay between Nrf2 and p65/RelA represents their competition for the binding site of the transcriptional coactivator CREB-binding protein (CBP), which both transcription factors require to propagate their signal [44, 76, 82]. Moreover, the overexpression of p65/RelA can limit the availability of CBP for complex formation with Nrf2, thus favoring increased NF-κB-driven gene expression [44]. Furthermore, it was shown that NF-κB can recruit the corepressor histone deacetylase 3 (HDAC3), causing local histone hypoacetylation of CBP or the Maf transcription factor protein (MafK), thereby potentially hampering Nrf2 signaling [76]. Collectively, the interplay of HO-1 and the IL-1 signaling pathway can occur through a variety of molecular interactions. We suggest that both NF-κB and HO-1 expression are essential for the EC response to inflammation and that an imbalance between the two pathways is associated with increasing inflammation. Our study complements the existing data by identifying IL-1β as a key regulator by suppressing HO-1 expression in BBB-ECs at a certain threshold, which results in increased autoimmune inflammation. Moreover, we show that EC-HO-1 is highly important to control EAE pathogenesis, mediated by the reduced expression of IL-1β-driven genes.

Pharmacological induction of HO-1 was reported to improve a variety of inflammatory diseases [3, 71], which suggests HO-1 as a potential target for therapeutic intervention also in MS. In this line, other studies suggested HO-1 to be beneficial for MS outcome, as this enzyme is increased within the CNS of MS patients [69]. Other studies could show that HO-1 expression in PBMCs of MS patients is reduced during disease progression and that during exacerbation of the disease there was a significant downregulation of HO-1 [24]. Furthermore, treatment with steroids increased the expression of HO-1, suggesting that HO-1 is protective against MS. The investigation of specific activators of the Nrf2/HO-1 pathway might be useful to improve anti-inflammatory mechanisms in MS. As a promising example, the Nrf2 activator dimethyl fumarate (DMF, Tecfidera^®^) is currently used to treat the relapsing–remitting form of MS, with rapid and sustained clinical and neurological efficacy [26, 33]. Importantly, DMF was shown to induce the downregulation of adhesion molecules, i.e. Vcam-1 and Icam-1 [20, 21], which was correlated with Nrf2 activation [26, 28, 67] and, therefore, supports the idea of a functional crosstalk between the NF-κB and Nrf2/HO-1 pathways. However, identification of a potent and specific Nrf2 activator or the targeting of its suppressors to treat MS and other inflammatory diseases requires further detailed investigations. We believe that a precise understanding of this crosstalk will help to target and manipulate the balance between Nrf2 and NF-κB signaling, which eventually represents a promising approach for improving MS therapies.

## Electronic supplementary material

Below is the link to the electronic supplementary material.

Supplementary file1 (DOCX 4677 kb)
