# Supplementary material for: Interleukin-1 promotes autoimmune neuroinflammation by suppressing endothelial heme oxygenase-1 at the blood–brain barrier
Source: Acta Neuropathol. 2020 Jul 11;140(4):549–67. doi: 10.1007/s00401-020-02187-x (PMC7498485; doi:10.1007/s00401-020-02187-x)
Supplement: Supplementary file 1 — Supplementary file1 (DOCX 4677 kb) [file 401_2020_2187_MOESM1_ESM.docx]

**SUPPLEMENTARY FIGURES**

Supplementary Figure 1


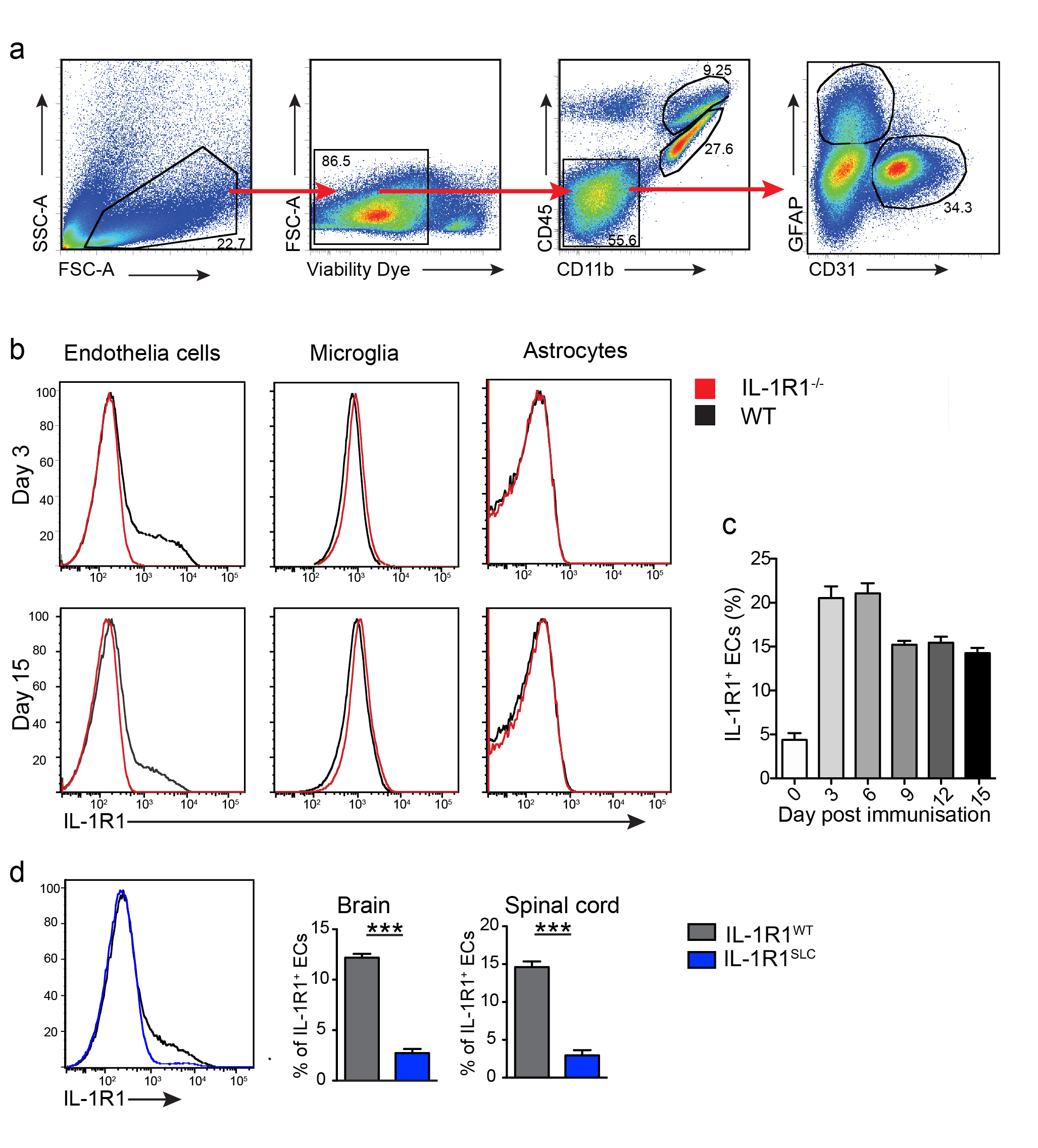


**Supplementary Fig. 1** IL-1R1 is expressed by BBB-ECs. Analysis of active EAE induction by immunization with MOG_35-55_/CFA and PTx injection of mice of the indicated genotypes. CNS tissue was isolated at indicated time points and single cell suspensions were analyzed by flow cytometry. **a** Representative gating strategy to identify living CD45^-^CD11b^-^CD31^+^ BBB-ECs, CD45^int^CD11b^+^ microglia and CD45^-^CD11b^-^GFAP^+^ astrocytes by flow cytometry. **b** Representative flow cytometry histograms of IL-1R1 expression by BBB-ECs, microglia and astrocytes, respectively, comparing cells isolated from WT or IL-1R1-deficient mice at different time points post immunization. **c** Frequencies of IL-1R1^+^ BBB-ECs at different time points post immunization. Data in **b** and **c** is representative for three individual experiments with at least n=4 per group. **d** Brain and spinal cord tissues were isolated from mice of the indicated genotypes at day 10 post immunization and single cell suspensions were analyzed by flow cytometry for IL-1R1 expression. Shown is a representative histogram of brain tissue and the quantification of the frequencies of IL-1R1^+^ ECs isolated from the indicated parts of the CNS. Data in **d** is representative for three individual experiments with at least n=3 per group. Data in **c** is shown as mean ± SEM and analyzed using 1-way ANOVA with Bonferroni’s post hoc test. Data in **d** is shown as mean ± SEM SEM and analyzed using two-tailed unpaired student’s t-test *** p<0.001

Supplementary Figure 2


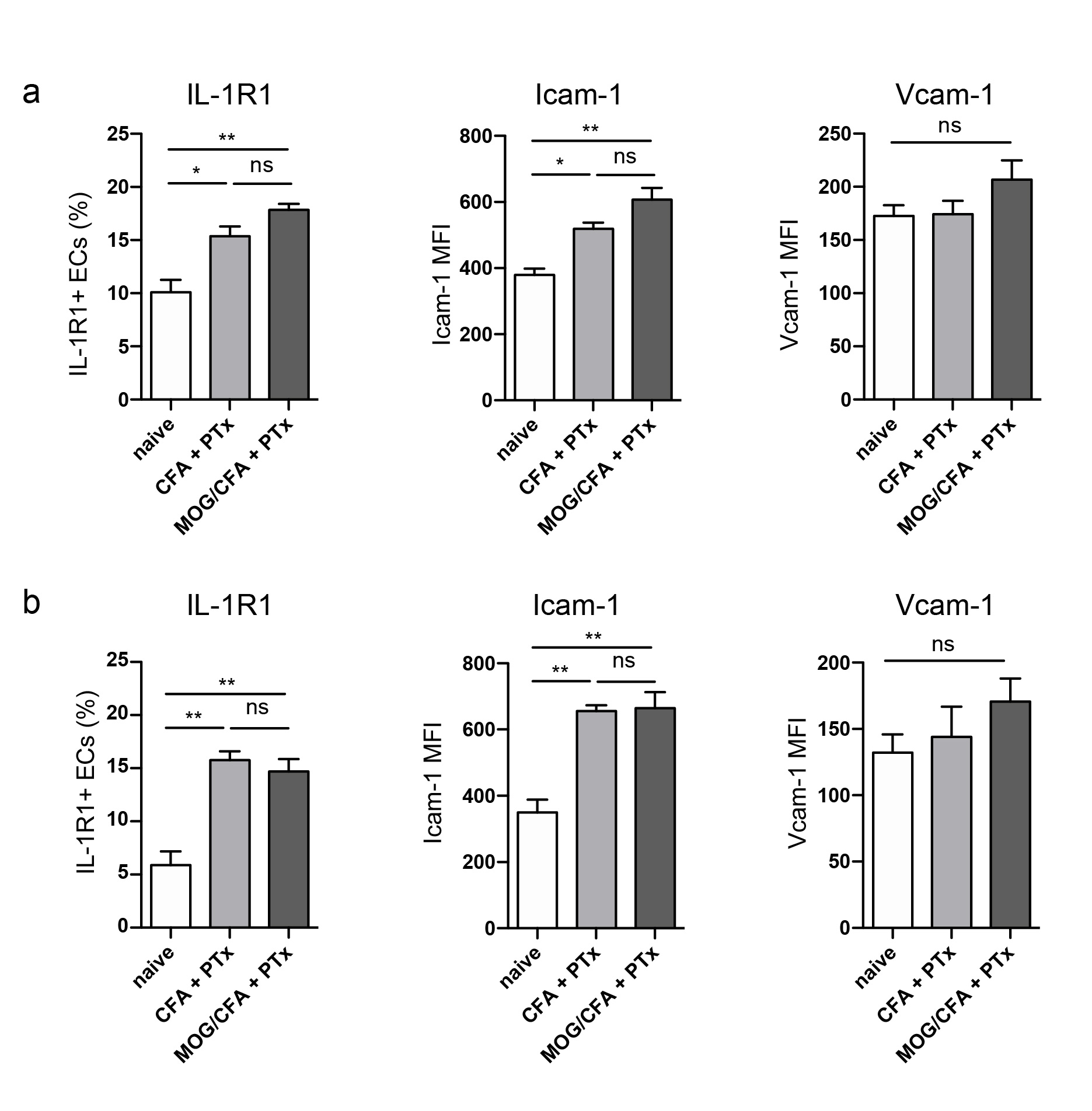


**Supplementary Fig. 2** IL-1R1 and Icam-1 expression by BBB-ECs is induced through CFA. Quantification of flow cytometry analysis showing the frequency of IL-1R1^+^ cells and mean fluorescence intensity (MFI) of Icam-1 and Vcam-1 expression in BBB-ECs (gated as CD45^-^CD11b^-^CD31^+^Ly6c^+^ living single cells) isolated from brain (**a**) and spinal cord (**b**) at day 3 post CFA +PTx or MOG/CFA+ PTx immunization. Data is shown as mean ± SEM and analyzed using one-way ANOVA. *p<0.05, ** p<0.01

Supplementary Figure 3


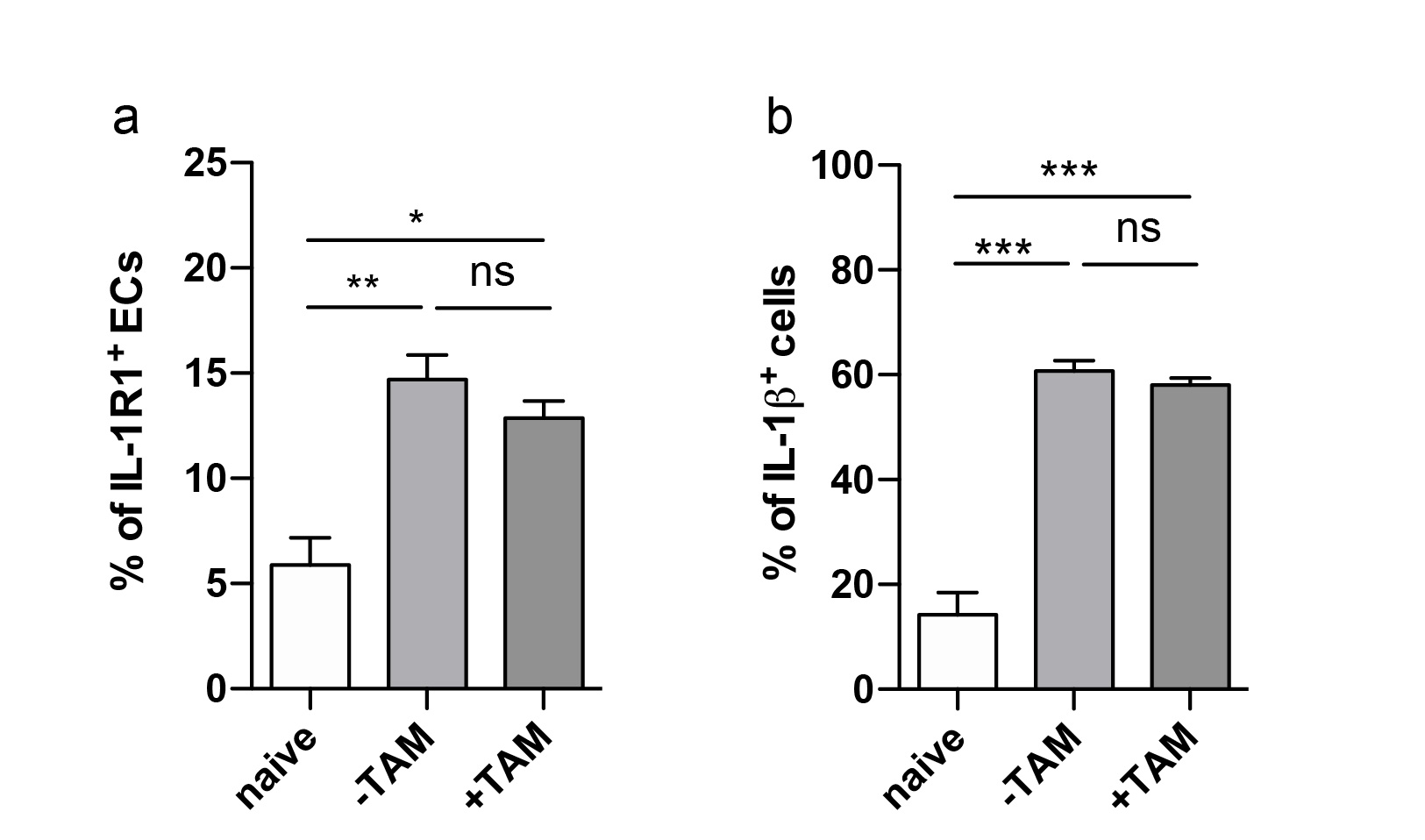


**Supplementary Fig. 3** Tamoxifen injection does not influence IL-1R1 expression or IL-1β production upon MOG/CFA immunization. Flow cytometric analysis showing the frequency of IL-1R1^+^ spinal cord ECs (gated as CD45^-^CD11b^-^CD31^+^Ly6c^+^ living single cells) (**a**) and frequency of IL-1β producing GR1^+^ splenocytes (gated as CD11b^+^ living single cells) stimulated for 4 h with 500 ng/mL LPS and 2 μM monensin (**b**). Wildtype mice were treated with 5 x 2 mg tamoxifen and immunized with MOG/CFA + PTx one week after final tamoxifen injection. Analysis was performed on day 3 post immunization. Data is shown as mean ± SEM (at least n=3 per group) and analyzed using one-way ANOVA *p<0.05, ** p<0.01, *** p<0.001

Supplementary Figure 4


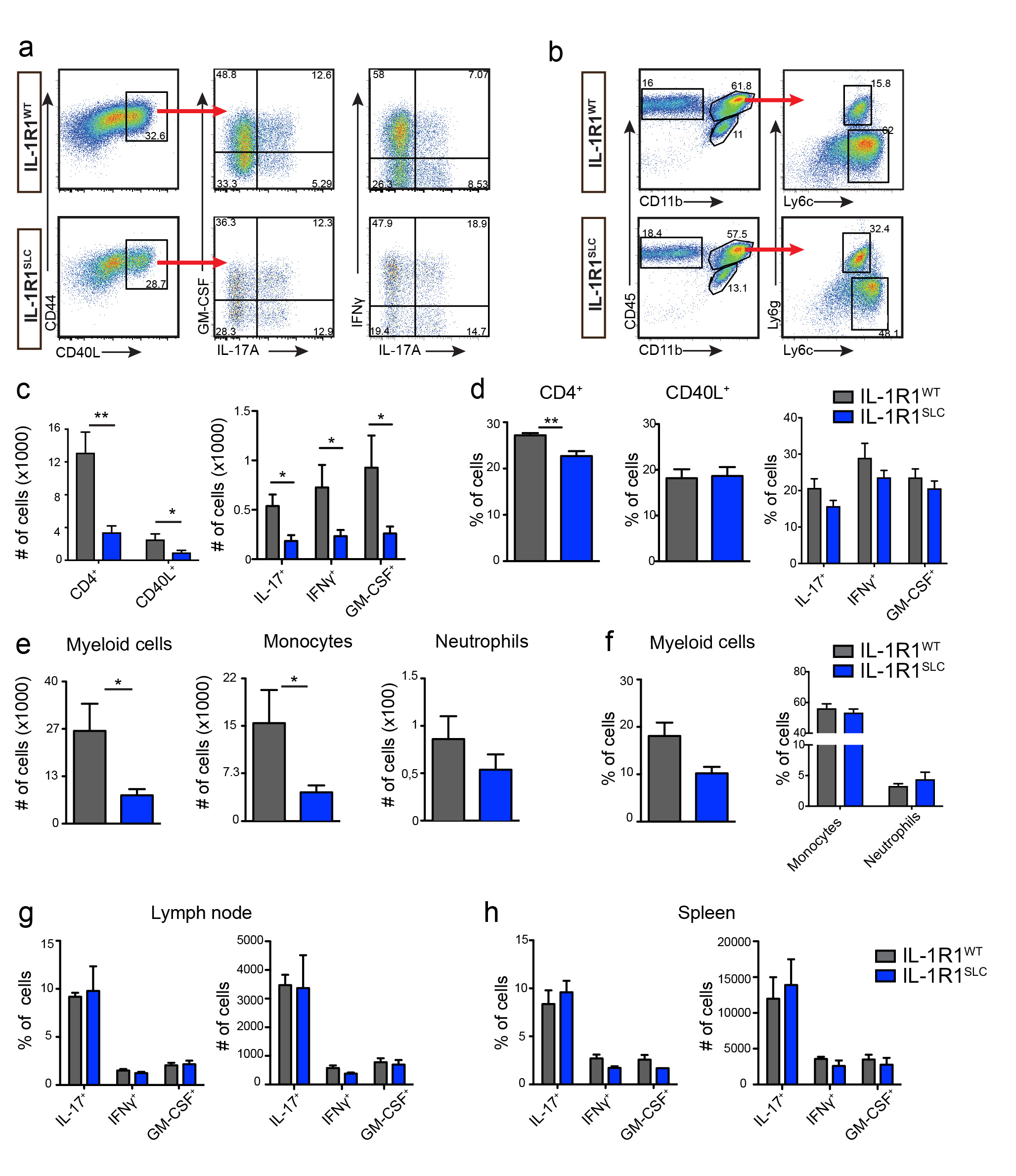


**Supplementary Fig. 4** BBB-EC IL-1 signaling promotes brain leukocyte migration. Analysis of brain tissue upon active EAE induction by immunization with MOG_35-55_/CFA and PTx injection of mice with the indicated genotypes. **a** Representative gating strategy to identify living CD90.2^+^CD4^+^CD44^+^CD40L^+^ MOG-specific T cells, which were further analyzed for their cytokine secretion. **b** Representative gating strategy for living CD45^+^CD11b^high^ myeloid cells, further gated on CD45^+^CD11b^high^Ly6c^high^ monocytes and CD45^+^CD11b^high^Ly6c^+^Ly6g^+^ neutrophils. **c**, **d** At peak of disease, brain tissue was isolated and single cell suspensions subjected to MOG antigen recall assays. Quantification of flow cytometry analysis shows the absolute cell numbers (**c**) and the frequencies (**d**) of the indicated T cell subsets. **e, f** At peak of disease, spinal cord tissue was isolated and single cell suspensions analyzed by flow cytometry. The quantification shows absolute cell number (**e**) and frequencies (**f**) of all living myeloid cells, further gated on monocytes and neutrophils. **g**, **h** At day 8 post immunization lymph nodes and spleen were isolated and single cell suspensions were subjected to MOG antigen recall assays. The quantification of flow cytometry analysis shows the frequencies and total cell numbers of MOG-specific T cells producing the indicated cytokines, as found in lymph nodes (**g**) and spleens (**h**) of IL-1R1^SLC^ and control mice. Data **a**-**h** is representative for at least three individual experiments with at least n=5 per group. Data in **c**-**h** is shown as mean ± SEM and analyzed using two-tailed unpaired student’s t-test. *p<0.05, ** p<0.01

Supplementary Figure 5


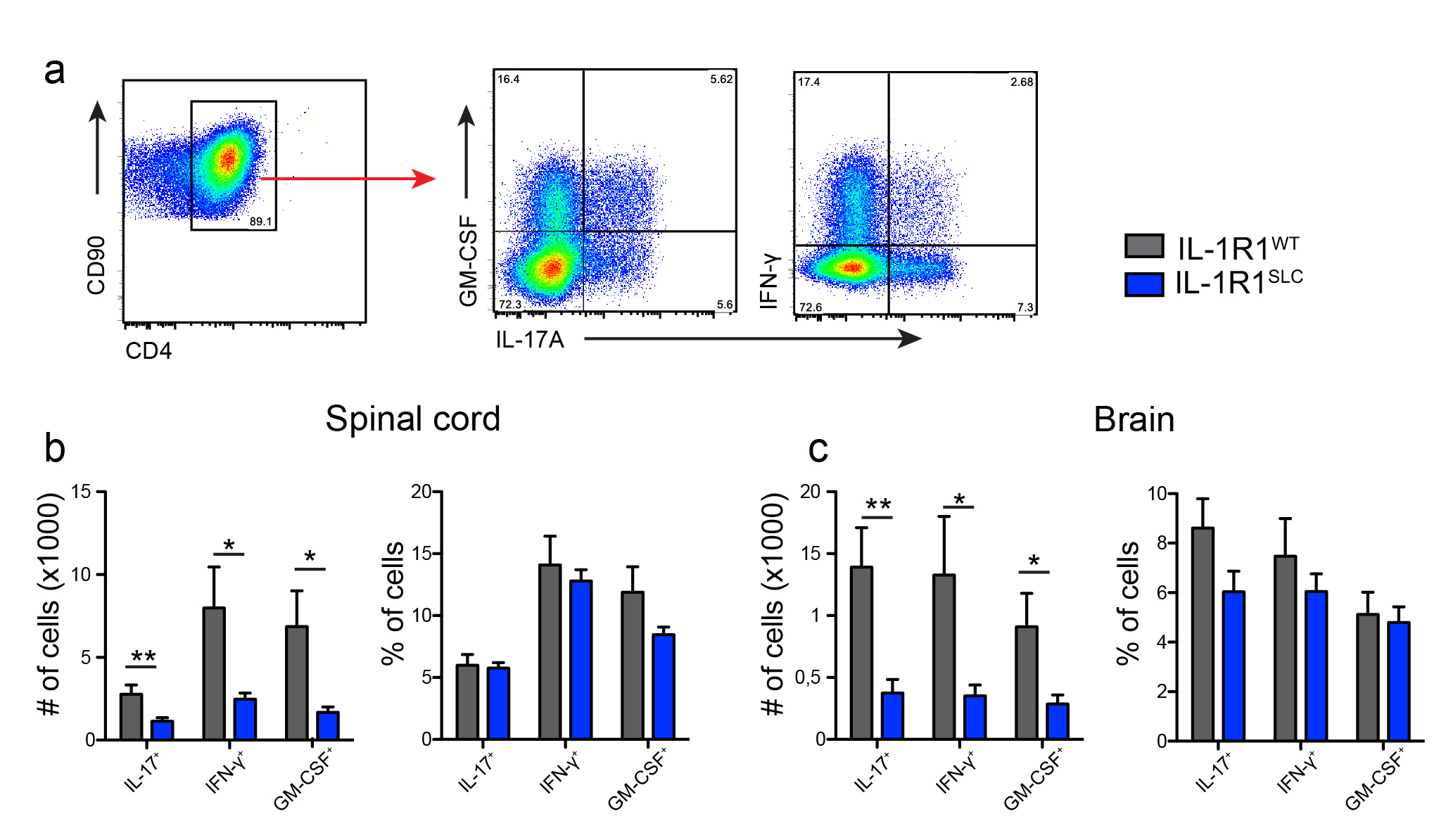


**Supplementary Fig. 5** BBB-EC IL-1 signaling promotes spinal cord and brain CD4^+^ T cell migration. Analysis of brain and spinal cord tissue upon active EAE induction by immunization with MOG_35-55_/CFA and PTx injection of mice with the indicated genotypes. **a** Representative gating strategy to identify living CD90.2^+^CD4^+^ T cells, which were further analyzed for their cytokine secretion at peak of disease. **b, c** Quantification of flow cytometry analysis shows the absolute cell numbers and the frequencies of the indicated T cell subsets from spinal cord (**b**) and brain (**c**) tissue. Data **a**-**c** is representative for at least three individual experiments with at least n=5 per group. Data in **b** and **c** is shown as mean ± SEM and analyzed using two-tailed unpaired student’s t-test. *p<0.05, ** p<0.01

Supplementary Figure 6

**
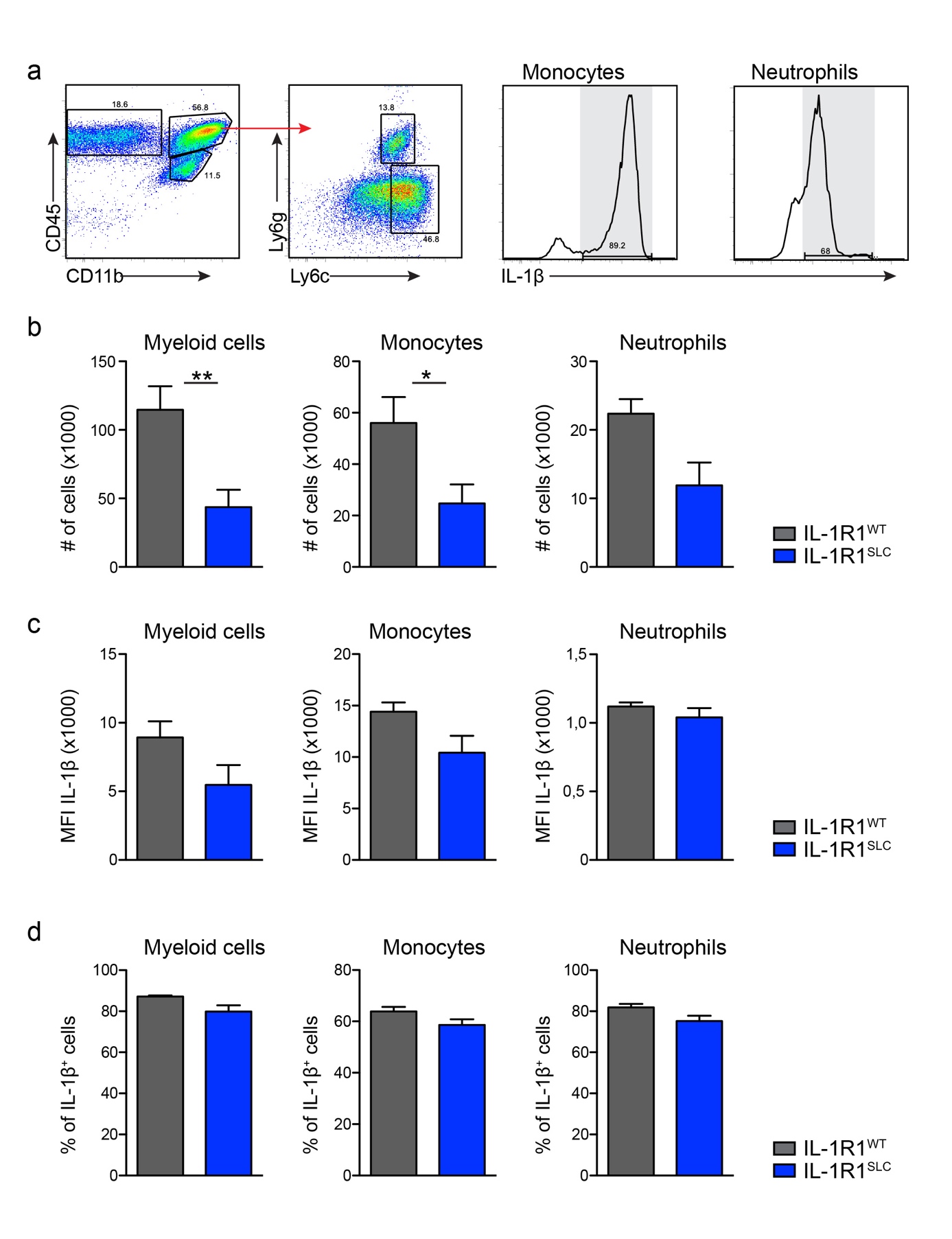
 Supplementary** **Fig. 6** EC-IL-1R1 expression does not impact the IL-1ß production by transmigrated myeloid cells **a** Representative gating strategy to identify living CD45^+^CD11b^high^ myeloid cells, CD45^+^CD11b^high^Ly6c^high^ monocytes and CD45^+^CD11b^high^Ly6c^+^Ly6g^+^ neutrophils, further analyzed for their IL-1β secretion. **a**-**d** For IL-1β detection isolated myeloid cells from spinal cord of EAE mice were incubated for 4h with 2 μM monensin and 500 ng/ml LPS. Flow cytometry showing cell counts (**b**) mean fluorescence intensity (MFI) (**c**) and frequency of IL-1β producing cells (**d**). Data in b-d is shown as mean ± SEM and analyzed using two-tailed unpaired student’s t-test ** p<0.01

Supplementary Figure 7


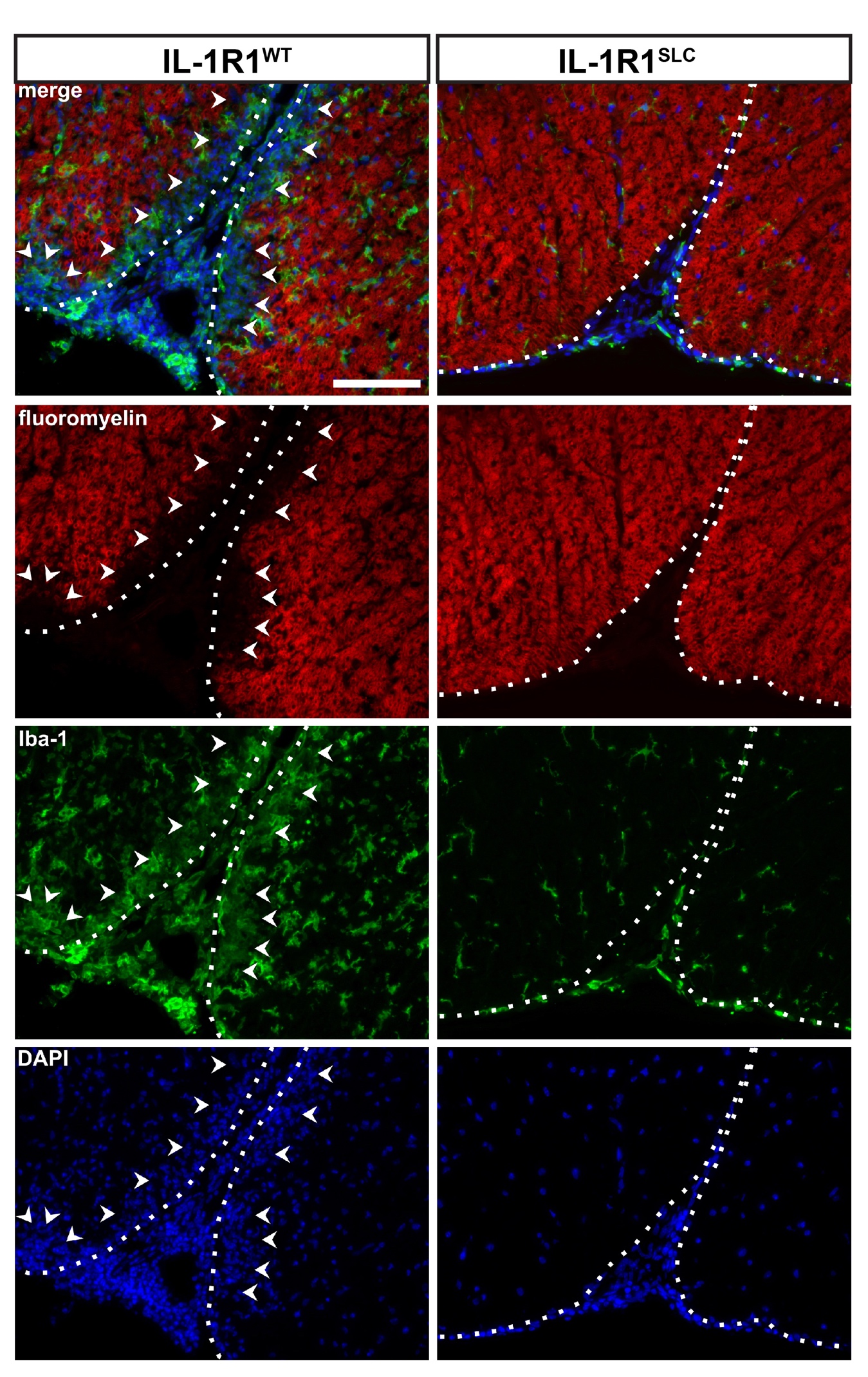


**Supplementary Fig. 7** BBB-EC IL-1 signaling promotes myelin damage and microglia activation. Immunofluorescence analysis of spinal cord tissue at EAE onset showing FluoroMyelin (red), Iba-1(green) and DAPI (blue) staining (scale bar = 100μm). White dots indicate the posterior horn of the spinal cord with cell infiltrates. Arrowheads show the area of demyelination.

Supplementary Figure 8


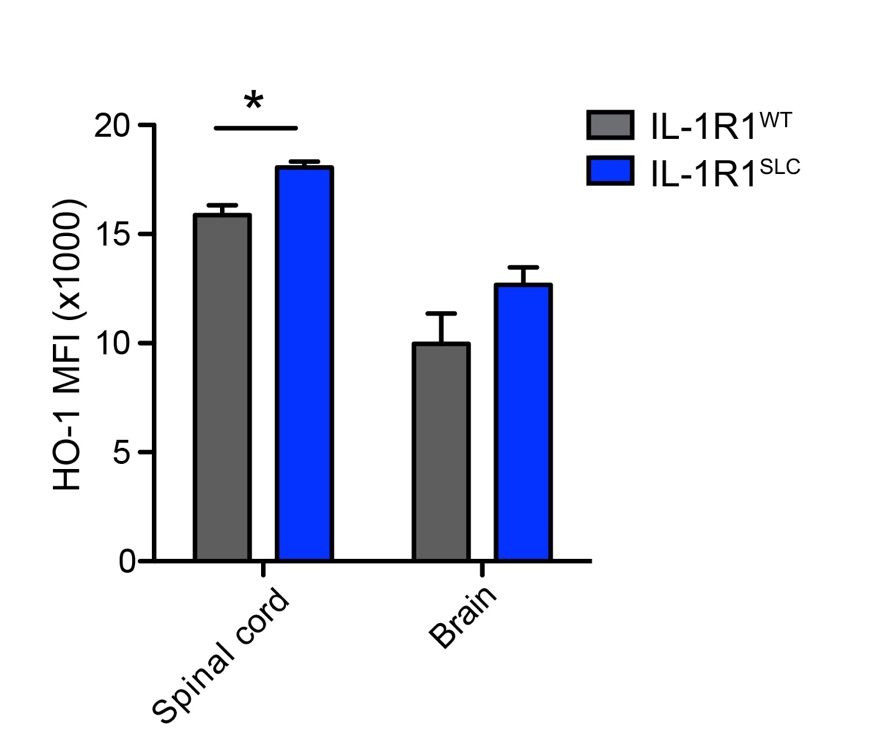


**Supplementary Fig. 8** HO-1 expression by BBB-ECs is increased in spinal cord of IL-1R1^SLC^ mice before onset of EAE. Flow cytometry analysis of BBB-ECs isolated from spinal cord and brain tissue, showing mean fluorescence intensity (MFI) of HO-1. Data is shown as mean ± SEM and analyzed using two-tailed unpaired student’s t-test *p<0.05

Supplementary Figure 9


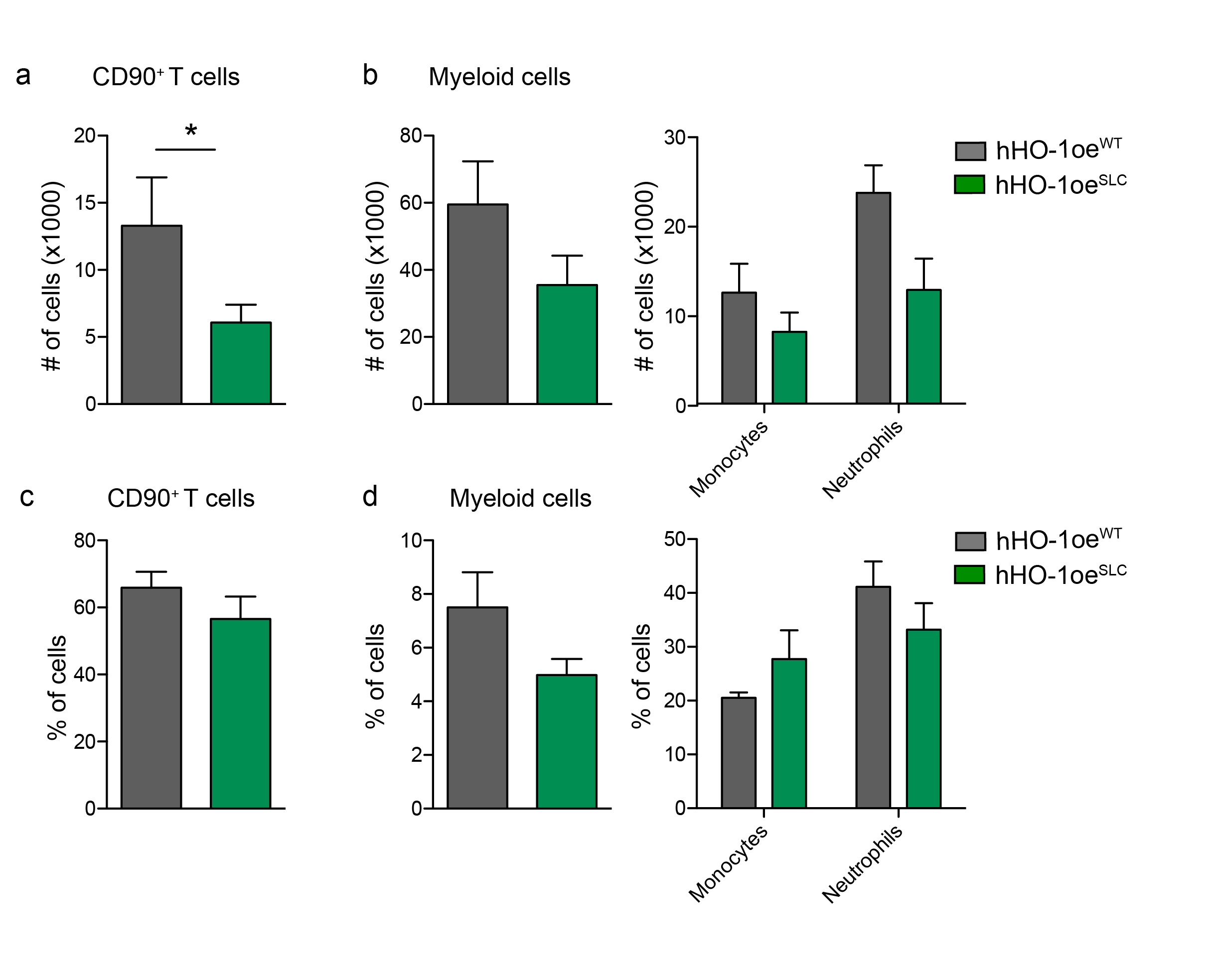


**Supplementary Fig. 9** HO-1 overexpression by BBB-ECs reduces leukocyte migration.

Single cell suspension isolated from CNS tissue of mice at day 10 post immunization were analyzed by flow cytometry for CNS-infiltrating leukocytes. Flow cytometry analysis was quantified and shows the absolute cell number (**a, b**) and frequency (**c, d**) of living CD45^+^CD90^+^ T cells (**a, c**) and of living CD45^+^CD11b^high^ myeloid cells, further gated on Ly6c^high^ monocytes and Ly6c^+^Ly6g^+^ neutrophils (**b, d**). Data is representative for at least two individual experiments with at least n=4 per group. Data is shown as mean ± SEM and analyzed using two-tailed unpaired student’s t-test. *p<0.05

Supplementary Figure 10


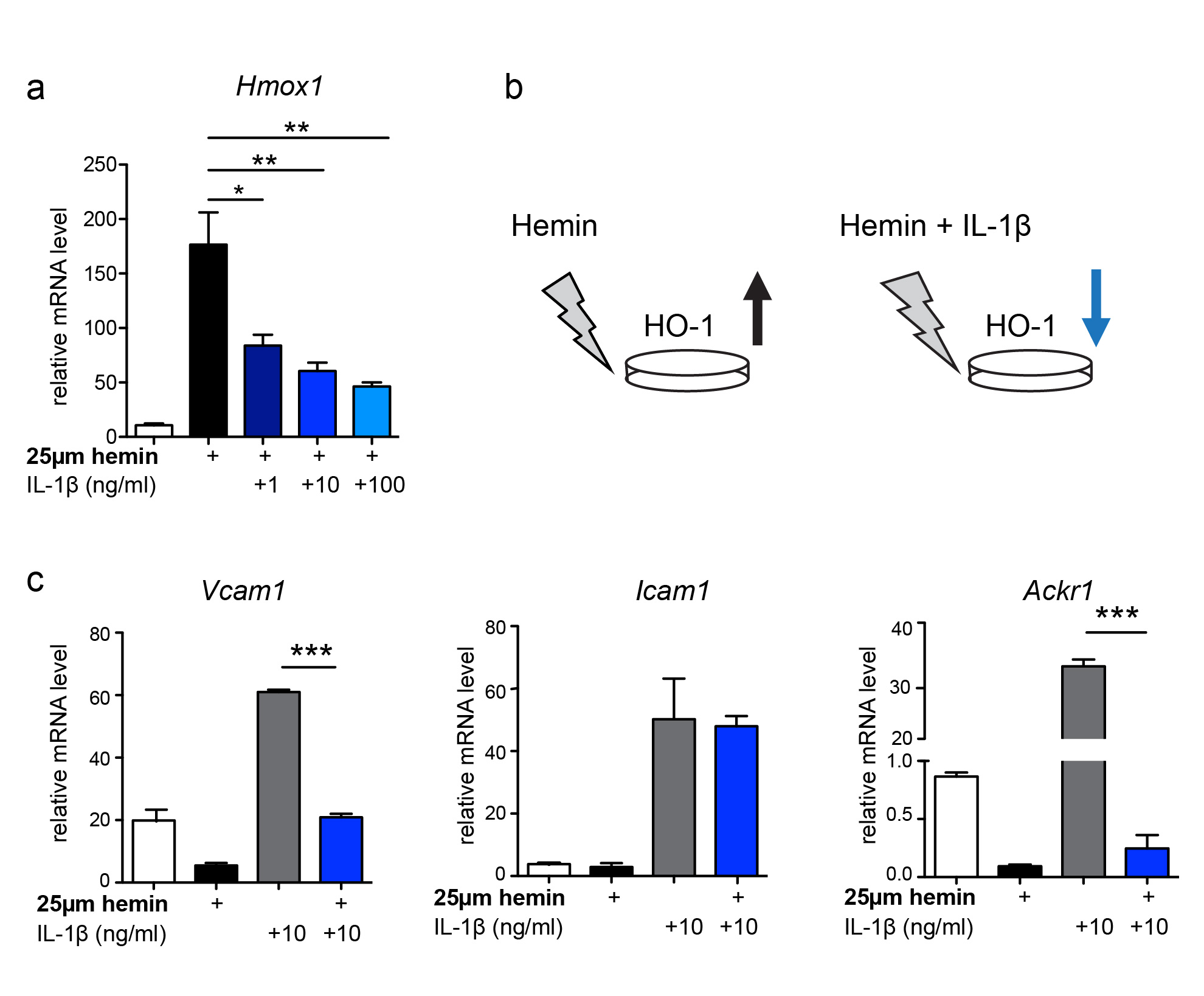


**Supplementary Fig. 10** EC-HO-1 and IL-1β signaling suppress each other and share a molecular crosstalk. **a-c** Primary mouse brain microvasculature endothelial cells (pMBMECs) were exposed to hemin, IL-1β or their combination at the indicated concentrations for 16 hr. **a** qRT-PCR analysis of *Hmox1* (HO-1) expression after the indicated treatment. **b** Schematic presentation of *Hmox1* (HO-1) expression upon different treatments. **c** qRT-PCR analysis of *Vcam1, Icam1 and Ackr1* (Darc) expression after the indicated treatments. Data in **a** and **c** is representative for three individual experiments with at least n=3 per group, and is shown as mean ± SEM and analyzed by using one-way ANOVA with Bonferroni’s post hoc test. *p<0.05, ** p<0.01, *** p<0.001
